# Dorsal anterior cingulate-brainstem ensemble as a reinforcement meta-learner

**DOI:** 10.1371/journal.pcbi.1006370

**Published:** 2018-08-24

**Authors:** Massimo Silvetti, Eliana Vassena, Elger Abrahamse, Tom Verguts

**Affiliations:** 1 Department of Experimental Psychology, Ghent University, Ghent, Belgium; 2 Institute of Cognitive Sciences and Technologies, National Research Council, Rome, Italy; 3 ‎Donders Institute for Brain, Cognition and Behaviour, Radboud University, Nijmegen, The Netherlands; 4 Basque Center on Cognition, Brain and Language, San Sebastián, Spain; 5 IKERBASQUE, Basque Foundation for Science, Bilbao, Spain; Oxford University, UNITED KINGDOM

## Abstract

Optimal decision-making is based on integrating information from several dimensions of decisional space (e.g., reward expectation, cost estimation, effort exertion). Despite considerable empirical and theoretical efforts, the computational and neural bases of such multidimensional integration have remained largely elusive. Here we propose that the current theoretical stalemate may be broken by considering the computational properties of a cortical-subcortical circuit involving the dorsal anterior cingulate cortex (dACC) and the brainstem neuromodulatory nuclei: ventral tegmental area (VTA) and locus coeruleus (LC). From this perspective, the dACC optimizes decisions about stimuli and actions, and using the same computational machinery, it also modulates cortical functions (meta-learning), via neuromodulatory control (VTA and LC). We implemented this theory in a novel neuro-computational model–the Reinforcement Meta Learner (RML). We outline how the RML captures critical empirical findings from an unprecedented range of theoretical domains, and parsimoniously integrates various previous proposals on dACC functioning.

## Author summary

A major challenge for all organisms is selecting optimal behaviour to obtain resources while minimizing energetic and other expenses. Evolution provided mammals with exceptional decision-making capabilities to face this challenge. Even though neuroscientists have identified a heterogeneous and distributed set of brain structures to be involved, a comprehensive theory about the biological and computational basis of such decision-making is yet to be formulated. We propose that the interaction between the medial prefrontal cortex (a part of the frontal lobes) and the subcortical nuclei releasing catecholaminergic neuromodulators will be key to such a theory. We argue that this interaction allows both the selection of optimal behaviour and, more importantly, the optimal modulation of the very brain circuits that drive such behavioral selection (i.e., meta-learning). We implemented this theory in a novel neuro-computational model, the Reinforcement Meta-Learner (RML). By means of computer simulations we showed that the RML provides a biological and computational account for a set of neuroscientific data with unprecedented scope, thereby suggesting a critical mechanism of decision-making in the mammalian brain.

## Introduction

Making the right decisions in uncertain and changing environments is at the heart of intelligent behavior [1,2]. To this purpose, the mammalian brain needs to integrate information from various dimensions of decisional space (e.g., reward expectation, costs estimation and effort exertion). Involvement of the medial prefrontal cortex (MFC), and in particular the dorsal anterior cingulate cortex (dACC), seems to be ubiquitous in experimental studies aimed at investigating this multidimensional integration [1]. Computational models suggested that many signals recorded in the dACC (e.g., error detection, error likelihood estimation and uncertainty) can be accounted for in terms of Reinforcement Learning (RL) operations [3]. Yet, dACC is also linked to adaptive control of (neuro)cognitive functions, like controlling physical or cognitive effort exertion, or regulating the right amount of neural plasticity as a function of environmental changes [4–7]. How these insights combine has so far remained elusive [8], resulting in an ongoing debate on the dACC functions and the neurobiogical basis of decision-making [8,9], (for a review see [10]).

Here we present a theoretical proposal on how the mammalian brain can optimize behaviour by simultaneously taking into account several dimensions involved in decision-making, and which role the dACC plays in this process. We start from the assumption, inherited from the RL domain, that decision-making is an optimization problem aimed at maximizing reward on the long term [11]. To pursue this optimization process, the mammalian brain needs to engage in *meta-learning*: it needs not only to optimally control ongoing behaviour (e.g. deciding whether or not to start chasing a prey), but also to learn how to control its own internal states that, in their turn, influence behavioural selection (e.g., deciding how much effort to invest in a chase). We propose that such meta-learning is carried out by a specific cortical-subcortical macrocircuit including the dACC, the brainstem catecholamine nuclei, ventral tegmental area (VTA) and locus coeruleus (LC), and their demonstrated bidirectional connections [12–18]. In this macrocircuit, RL principles are exploited to select appropriate behavioural responses and to modulate its own internal states via dopamine (DA, synthetized by VTA) and norepinephrine (NE, synthetized by LC) neuromodulation. We implemented this theoretical proposal in a novel computational model coined the Reinforcement Meta Learner (RML), modeling the dACC, the VTA, and locus coeruleus LC. Like in earlier RL models, the dACC in the RML computes the values of specific stimuli and actions to achieve adaptive behavior. However–and unlike in earlier models–dACC internal dynamics is modulated by catecholamines via recurrent interaction between the dACC itself and the brainstem nuclei.

It is worth stressing that a single fixed parameter set is used in the RML to simulate empirical findings from an unprecedented range of theoretical domains. This demonstrates that the RML provides a viable model for dACC functioning and a first potential step toward theoretical unification across these domains, inspiring new perspectives on the biological and computational foundations of decision-making in the mammalian brain.

### Paper structure

In the next two subsections of the Introduction we describe qualitatively the computational principles of the RML (The RML: General description) and the main novelties introduced by the model (The RML: Innovations). In the subsequent Results section, we describe the experimental paradigms we used to test the RML and the results, together with domain-specific discussion paragraphs. Next, in the domain-general Discussion section we broadly frame and connect the results, comparing our model with other models from recent literature (Relationships to Other Models). We also propose future experimental paradigm to test RML predictions (Experimental Predictions), including possible applications to translational research, and we describe some limitations of our work (Limitations). Finally, in the Methods section we provide the full mathematical description of the RML.

### The RML: General description

**Fig 1:**
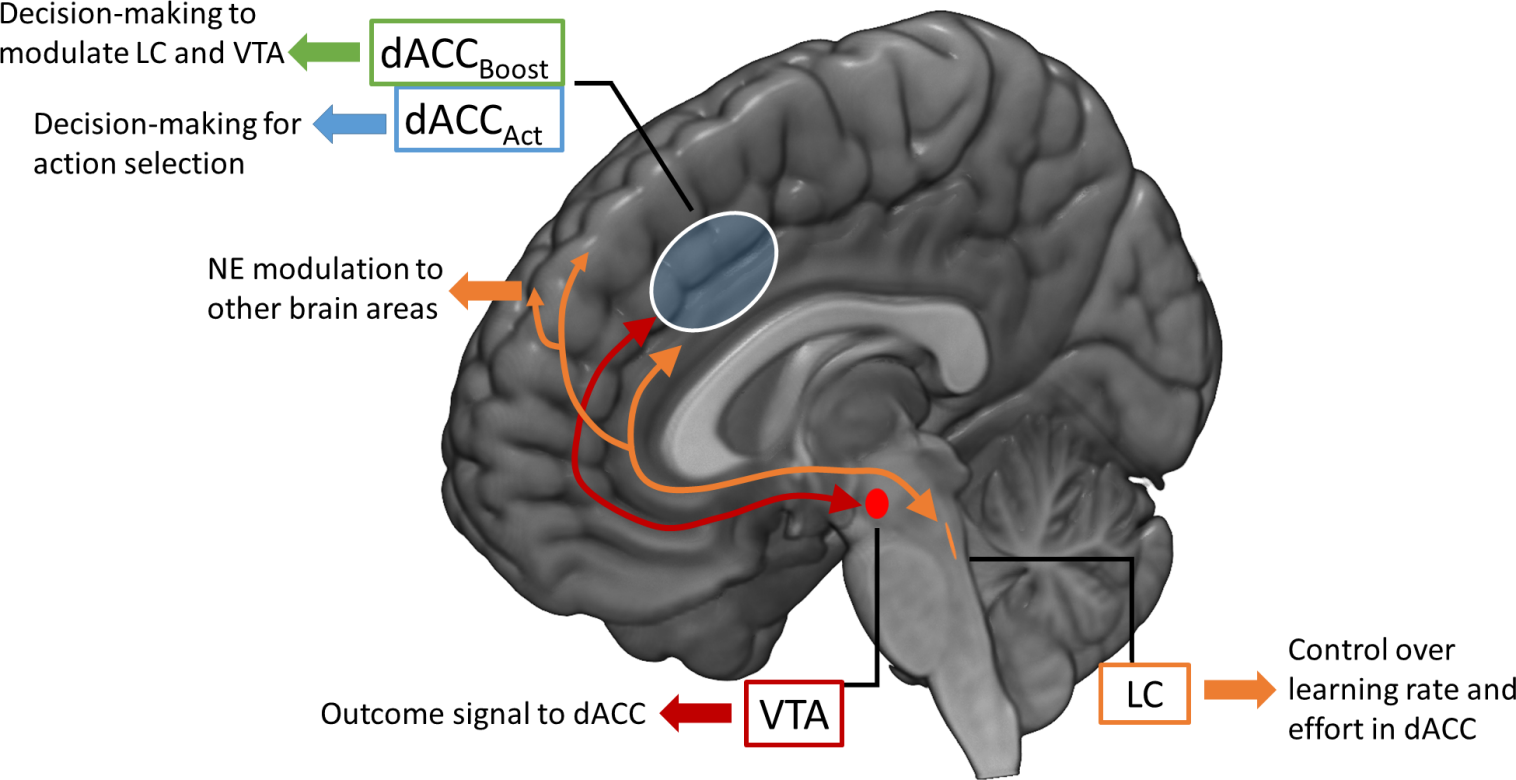
RML overview with neuroanatomical mapping. The RML consists of a state-action selection dual system (dACC_Act_ and dACC_Boost_), based on RL algorithms, and a parameter modulation system via catecholamine release (VTA and LC) that are in constant interaction. Finally, the RML can be connected to an external neural model (e.g. a fronto-parietal network) and part of the LC output (NE) can be used to modulate its activity while the entire system (RML + external model) is interacting with the environment.

At the basis of our model is the idea that a macrocircuit involving dACC-VTA-LC represents a core computational unit for optimizing both behaviour and internal states that modulate behaviour itself (meta-learning). Fig 1 represents an overview of the RML architecture. The RML dynamics is based on two inter-related loops connecting four computational modules: dACC_Boost_, dACC_Act_, VTA, and LC. An *external loop* represents the interaction between the dACC modules and the environment, while an *internal loop* covers the interaction between the dACC modules and the brainstem nuclei (VTA and LC; orange and red bidirectional arrows in Fig 1). This double loop structure is aimed at optimizing performance (i.e., maximizing reward) while minimizing two different types of costs: the costs of motor actions (external loop; e.g. the metabolic cost of climbing a stair), and the boosting costs of neuromodulators release (internal loop; e.g. the cost of neurotransmitters depletion). Connectivity and functional studies corroborate the hypothesis underlying this architecture, because they show that there is an anatomical overlap between the midfrontal sub-region related to the meta-learning processes discussed above, and the midfrontal sub-region maximally connected with both LC and VTA nuclei [13,18–21], both located within the dACC area.

In the RML, the dACC plays the role of a performance monitoring system, which compares expectations about environmental states and executed actions with environmental outcomes (cf. [22]). Discrepancies between expectations and outcomes generate prediction error (PE) signals (Figure G in S2 File), which are used to update the expectations themselves [3]. This monitoring process is at the basis of two dACC modules, and in substantial agreement with the experimental literature (see [3] for a review). One dACC module (dACC_Act_ in Fig 1) receives environmental *states* and selects *actions* directed toward the external environment (part of the *external loop*). Although value-based action selection involves also other subcortical and cortical structures (e.g. the dorsolateral prefrontal cortex, DLPFC), here we frame both value estimation and action selection within the dACC for both modeling parsimony and because also the MFC, with its motor components, plays an important role in action selection (see [3] for a review).

A second dACC module (dACC_Boost_ in Fig 1) receives environmental states and consequently modulates (that is, *boosts*) the release of catecholamines from the brainstem nuclei LC and VTA (part of the *internal loop*). Catecholamines, in turn, control the internal dynamics of the dACC in real time (i.e. while the RML is interacting with the environment), by modulating the magnitude of *reward* signals (by VTA module) and the amount of *effort* (by LC module) that the RML exerts to execute a task. Although the dACC_Boost_ module is the main responsible for catecholaminergic modulation, the dACC_Act_ module, too, is in recurrent interaction with the brainstem nuclei, providing the VTA with a reward prediction signal. The latter is used by the VTA to compute *non-primary rewards*, which are sent back to the dACC_Act_ (like in a TD-learning algorithm; [23]) allowing the system to learn complex tasks without the immediate availability of primary rewards (higher-order conditioning).

Importantly, both dACC modules have dynamic *learning rates* (λ), ensuring that knowledge is updated only when there are relevant environmental changes (volatility). Learning rate adaptation emerges from the interaction between the LC and both dACC modules. Each dACC module feeds the LC with reward prediction and PE signals, while the LC analyzes these “raw data” from the cortex (approximating a Bayesian learner), estimating volatility and adjusting the modules’ learning rate as a consequence.

Finally, the RML can be connected to other neural models (e.g. a visuo-spatial working memory model, see Simulation 2c). This allows the effort-related signal from the LC to modulate processing in other brain areas for performance optimization (Fig 1, orange arrows; see Methods for details).

### The RML: Innovations

In this section we briefly introduce the main theoretical novelties of the RML. For a more detailed analysis we address the reader to the Discussion section, where we also relate the RML in detail to previous models, describe explicit experimental predictions that derive from the model, and speculate on the potential application of the RML to translational research.

The RML is an autonomous agent able to near-optimally adapt to a diverse range of environments and tasks, with no need of task-specific parameters setting: across all the reported simulations the RML autonomously controlled its internal dynamics as a function of the environmental challenges, with no offline parameters optimization or human intervention (i.e. one parameter set was used for all the simulations). From here, four major novelties can be identified.

#### Meta-learning via recurrence with brainstem neuromodulators

The RML internal loop (dACC-VTA-LC) allows meta-learning of optimal modulation over learning rate, reward, and effort. Due to this interaction, the RML achieves autonomous flexibility to manage changing demands in cognitive control. To the best of our knowledge, this is the first computational theory on how neuromodulators are controlled during task execution and how these can influence behavioural performance. This also implies that several decision problems that in earlier work were tackled via hierarchical models (e.g. hierarchical models for effort modulation [24] or hierarchical Bayesian models [4]) are solved here by the RML loops that have no intrinsic hierarchical structure itself (cf. simulations 1 and 2a-c, and “Relationships to Other Models” section). Our proposal adds a novel theoretical perspective with the aim of being complementary rather than alternative to hierarchical models.

Control on other neural circuits. The external loop of the RML can be used not only to drive optimal external behavior, but also to drive optimization of brain networks beyond (thus external to) the dACC-VTA-LC circuit. Specifically, the LC module (under the influence of the dACC) generates control signals (based on task demands) that can modulate (e.g. gain modulation) the activity of other neural modules. This simulates how the dACC can exert cognitive control over other brain areas, via catecholaminergic modulation. For example, we simulated how the fronto-parietal network in working memory tasks can be optimally modulated by LC NE release, thanks to the dialogue between the dACC and the brainstem nuclei (cf. Simulation 2c). Thus, the RML can generate cognitive control signals for improving the performance of different, independently designed and published models.

#### Comprehensive understanding of DA dynamics

In the RML, the typical DA dynamics as recorded from the VTA during conditioning tasks (e.g. PE coding and DA shifting from reward onset to cue onset) emerges from the interaction between dACC and VTA–in contrast with the classical view representing the VTA itself as the main source of PE and temporal difference (TD) signals (e.g. [25,26]). This mechanism (see also [22]) is based on a large amount of empirical data identifying the dACC as a major source of PE signals (see [3] for a review); it is here integrated within a comprehensive theory on the cortical origin of the DA dynamics and what could be its computational role in decision-making.

#### Mechanism underlying intrinsic motivation

As the dopaminergic reward signals from midbrain to dACC are modulated by the dACC itself (based on task demand), the RML implements a computational hypothesis about the mechanisms behind intrinsic motivation [27], i.e. on how the mammalian brain can energize behavior in a way that is independent from the immediate availability of primary rewards (cf. Simulations 3a-b).

## Results

Here we present the results on both neural and behavioural dynamics of the RML in six key experimental paradigms selected from lower and higher cognitive decision-making domains. We show how the RML can provide a unified framework to explain experimental data from a set of decision-making contexts to which dACC and catecholamines are often related, namely optimal decision-making in uncertain and volatile conditions (Simulation 1), and optimal control of both physical and cognitive effort exertion (Simulations 2a-c). Finally, we generalize our findings to a domain where the dACC is typically not discussed, yet very important in decision-making, i.e. modulating intrinsic motivation to learn complex tasks without the immediate availability of primary rewards (higher-order conditioning; Simulations 3a-b). As the RML represents a generalization of previous RL models of MFC functions (the RVPM [22], and the PRO model [28]), it can reproduce also all the experimental findings simulated by those models (e.g. error detection and error likelihood estimation). This extends the RML results to an even wider domain of experimental paradigms.

To mimic standard experimental paradigms as closely as possible, we repeated each simulation only 12 times (i.e., 12 simulated subjects). This verified that the model can generate a large effect size of results. Obviously, p-values (but not effect sizes) improved when running more simulated subjects. Further details on simulations methods can be found in the Supporting Information in S1 File.

### Simulation 1: Learning rate optimization

Adaptive control of learning rate is a fundamental aspect of cognition. Humans can solve the tradeoff between stability and plasticity in a (near) Bayesian fashion [4,29], distinguishing between variability due to noise versus variability due to actual changes of the environment; thus they can increase the learning rate only when volatility is detected [30,31]. At the neural level, a currently unexplained dissociation exists between dACC and LC activity, recorded during decision-making tasks where uncertainty due to noise and uncertainty due to volatility were systematically manipulated. The LC activity (and thus NE release) has been shown to track specifically volatility [30,32,33], while the results about the dACC role in volatility estimation are less consistent. Indeed, while in the seminal study by Behrens et al. [4], the dACC was hypothesized to track volatility, more recent study suggested that dACC activity in volatile environments are driven rather by PE coding, rather than specifically by volatility estimation [21]. These empirical findings seem to attribute different roles to LC and dACC in uncertainty coding, without providing a computational rationale for their functional specialization.

In this simulation, we will investigate to what extent the model accounts for human adaptive control of learning rate at both behavioural and neural levels, and whether it can explain the dACC/LC dissociation.

#### Simulation methods

**Fig 2:**
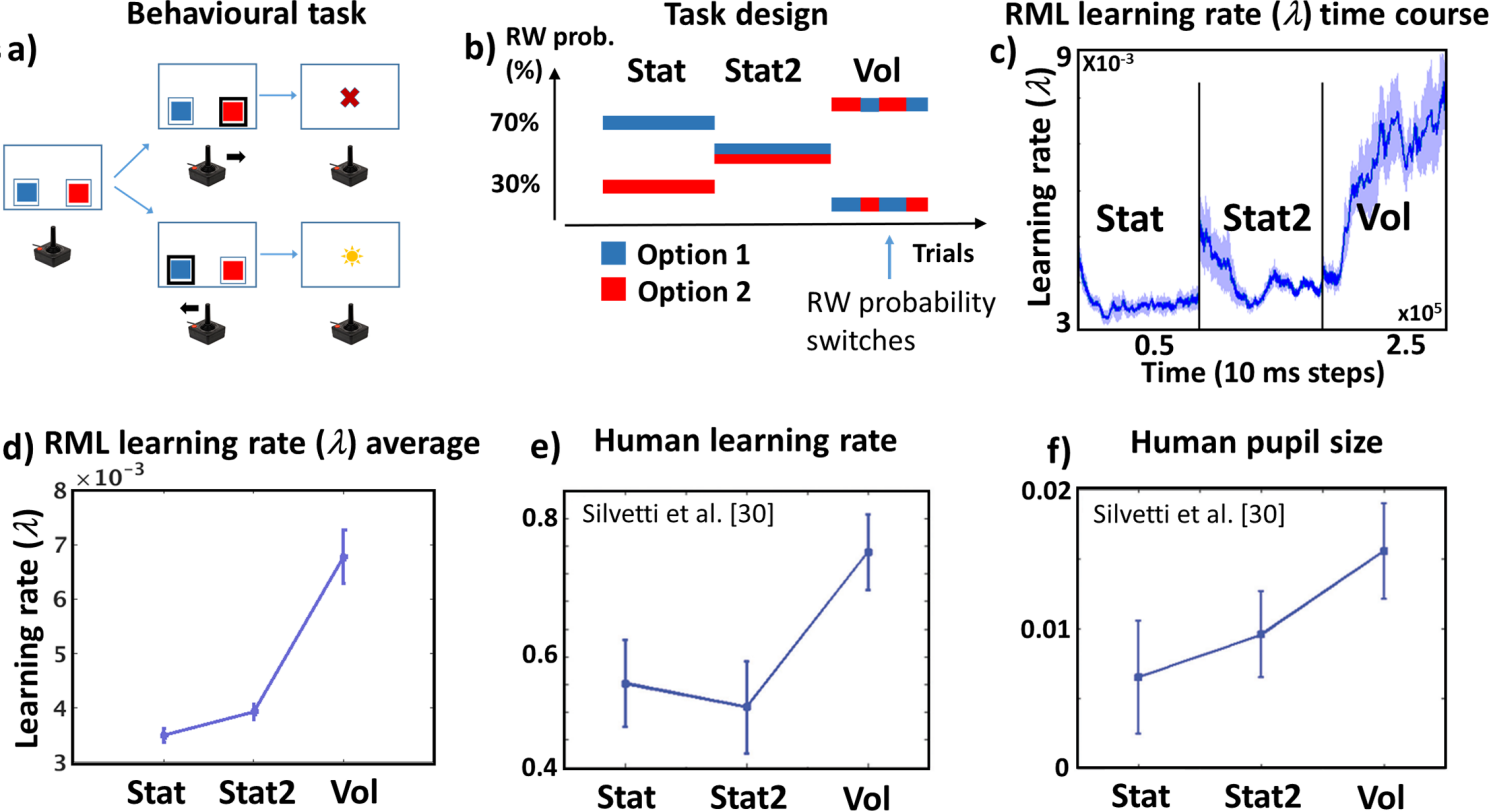
Simulation 1: Methods and results. **a)** The task (2-armed bandit) is represented like a binary choice task (blue or red squares), where the model decisions are represented as joystick movements. After each choice, the model received either a reward (sun) or not (cross). **b)** Example of task design with time line of statistical environments (order of presentation of different environments was randomized across simulations). The plot shows reward probability linked to each option (blue or red) as a function of trial number. In this case the model executed the task first in a stationary environment (Stat), then in a stationary environment with high uncertainty (Stat2), and finally in a volatile (Vol) environment. **c)** Learning rate (*λ*) time course (average across simulations ± s.e.m.). As the order of statistical environments was randomized across simulations, each simulation time course was sorted as Stat-Stat2-Vol. **d, e)** Average ∠ (across time and simulations) as a function of environmental volatility (± s.e.m.) in the RML (d) and humans (**e;** modified from: [30]). **f)** human pupil size (proxy of LC activity [34–36]) during the same task.

We administered to the RML a 2-armed bandit task in three different stochastic environments (Fig 2A and 2B). The three environments were: stationary environment (Stat, where the links between reward probabilities and options were stable over time, either 70 or 30%), stationary with high uncertainty (Stat2, also stable reward probabilities, but all the options led to a reward in 60% of times), and volatile (Vol, where the links between reward probabilities and options randomly changed over time). We administered a total of 432 trials equally distributed between the three statistical environments. We assigned higher reward magnitudes to choices with lower reward probability, to promote switching between choices and to make the task more challenging (cf. [4]). Nonetheless, the value of each choice (probability × magnitude) remained higher for higher reward probability (see Table B in S1 File), meaning that reward probability was the relevant variable to be tracked. A second experiment, where we manipulated reward magnitude instead of reward probability led to very similar results (see Simulations S1 and S3 in S2 File).

#### Simulation results and discussion

The RML performance in terms of optimal choice percentages was: Stat = 66.5% (± 4% s.e.m.), Vol = 63.6% (± 1.4% s.e.m.). For Stat2 condition there was no optimal choice, as both options led to reward in 60% of all trials. Importantly, the model successfully distinguished not only between stationary (Stat) and volatile (Vol) environments, but also between stationary-uncertain (Stat2) and Vol, increasing the learning rate (computed in the LC module) exclusively in the latter (Fig 2C and 2D). Indeed, there was a main effect of volatility on learning rate *λ* (F(2,11) = 29, p < 0.0001). Post-hoc analysis showed that stationary conditions did not differ (Stat2 > Stat, t(11) = 1.65, p = 0.13), while in the volatile condition learning rate was higher than in stationary conditions (Vol > Stat2, t(11) = 5.54, p < 0.0001; Vol > Stat, t(11) = 5.76, p < 0.0001). Hence, the interaction between dACC and LC allows disentangling uncertainty due to noise from uncertainty due to actual environmental changes [30,31] promoting plasticity (high learning rate) when new information must be acquired (condition Vol), and stability (low learning rate) when acquired information must be protected from noise (conditions Stat and Stat2). This mechanism controls learning rates in both the dACC_Act_ and the dACC_Boost_ modules, thus influencing the entire RML dynamics.

**Fig 3:**
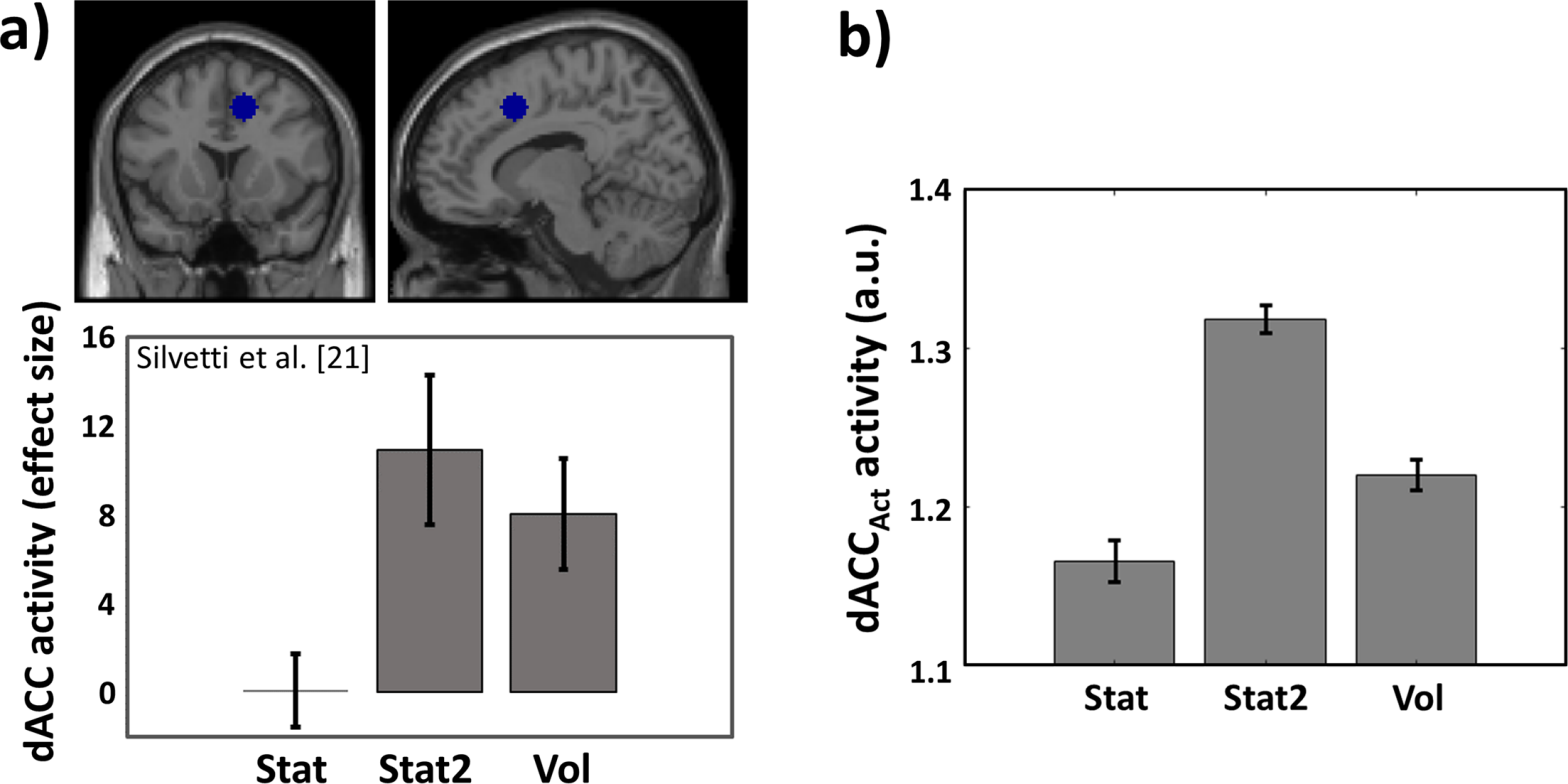
Simulation 1: Results comparison with fMRI data. **a)** Outcome-locked activation of human dACC (with 90% CI, extracted from the ROI indicated by the blue sphere; MNI: [12,14,44]) in a RL task executed during fMRI scanning. Data extracted by WebPlotDigitizer from Fig 4 in ref. [21]. The ROI is a local maximum within the cluster with the highest z value. The task was performed in the same three environments we used in our simulations. dACC activity peaked in Stat2 and not in Vol condition (Stat2 > Vol, p < 0.05), indicating responsiveness to overall uncertainty (i.e. PE) rather than to volatility (see ref. [21] for further details) **b)** dACC_Act_ average activity (sum of PE units activity ± s.e.m.; see Eq 1 and Equations S3-S4 in S1 File) as a function of environmental uncertainty. Differently from the LC, the dACC_Act_ is maximally active in stationary uncertain environments (Stat2), indicating that due to PE computation, dACC_Act_ (like the human dACC) codes for overall uncertainty rather than for volatility.

Differently from the LC, dACC_Act_ showed a maximal activation in the Stat2 environment (uncertain) rather than in the volatile environment (Fig 3B). This dissociation is due to the different roles played by dACC and LC in learning rate control (see Eq 5A in Methods). Indeed, while dACC modules compute reward expectation and PE, the LC performs approximate Bayesian analysis on those signals to compute optimal learning rate. For this reason, the dACC is more responsive to overall environmental uncertainty (expressed by average PE), while LC selectively responds to volatility. As mentioned before, this dissociation between the LC and the dACC dynamics simulated by the RML were found also in humans. Indeed, in the same task, humans increased both learning rate and LC activity only in Vol environments [30] (Fig 2E and 2F). Moreover, during a RL task executed in the same three statistical environments used in this simulation, the human dACC activity peaked for the Stat2 environment, suggesting that activity of human dACC is dominated by PE rather than by explicit estimation of environmental volatility [21] (Fig 3A).

### Simulation 2: Controlling physical and cognitive effort

A long list of experimental results indicates that DA and NE neuromodulators are not only crucial for learning environmental regularities, but also for exerting cognitive control [37–41]. Although these mechanisms have been widely studied, little is known about how the brainstem catecholamine output is controlled to maximize performance [31,42,43], and how the dACC is involved in such a process. In this section, we describe how the dACC_Boost_ module learns to regulate LC and VTA activity to control effort exertion, at both cognitive and physical level [19,44,45]. In Simulation 2a, we test the cortical-subcortical dynamics in experimental paradigms involving decision-making in physically effortful tasks, where cost/benefit trade off must be optimized [46–48]. In Simulation 2b, we show how the LC can provide a NE signal to external neural modules to optimize cognitive effort [19,20] allocation and thus behavioural performance in a visuo-spatial working memory (WM) task. In both simulations, we also test the RML dynamics and behaviour after cortical and subcortical lesions.

### Simulation 2a: Physical effort control and decision-making in challenging cost/benefit trade off conditions

Deciding how much effort to invest to obtain a reward is crucial for human and non-human animals. Animals can choose high effort-high reward options when reward is sufficiently high [46,47]. The impairment of the mesolimbic DA system strongly disrupts such decision-making [46,47]. Besides the VTA, experimental data indicate also the dACC as having a pivotal role in decision-making in this domain [19,20,48–50] (see also[51] for a review). In this simulation, we show how cortical-subcortical interactions between the dACC, VTA and LC can drive optimal decision-making when effortful choices leading to large rewards compete with low effort choices leading to smaller rewards. We thus test whether the RML can account for both behavioral and physiological experimental data from humans and nonhuman animals. Moreover, we test whether simulated ACC lesion or DA depletion can replicate the disruption of optimal decision-making, and, finally, how effective behaviour can be restored. Simulation results will be compared with behavioural data from rodents ([47], see also Simulation 2a in S1 File), and with physiological data from nonhuman primates [35] and humans [44]. Rodent data from Walton et al. [47] were chosen for comparison to study how the cost-benefit trade-off could be affected by ACC damage and by DA lesion and how behavioural performance could be partially recovered with environmental intervention (Simulation 2b). We express the caveat that DA depletion studies in the literature we cited ([46,47], to compare with RML performance) either deplete DA systemically, or are focused more on the mesolimbic-accumbens path than on DA afferents to the medial prefrontal cortex. Our assumption that mesolimbic DA lesion affects dACC functioning is neurophysiologically sound, because functional and anatomical connectivity indicates strong nucleus accumbens (NAc)—dACC connectivity [12,13,52,53], probably contributing to convey reward-related information to the dACC. For this reason, lesioning the NAc may also disrupt the information flow from VTA to the dACC. Moreover, our simulations lead to the experimental prediction that DA lesion to dACC generates effects similar to mesolimbic DA lesions.

#### Simulation methods

**Fig 4:**
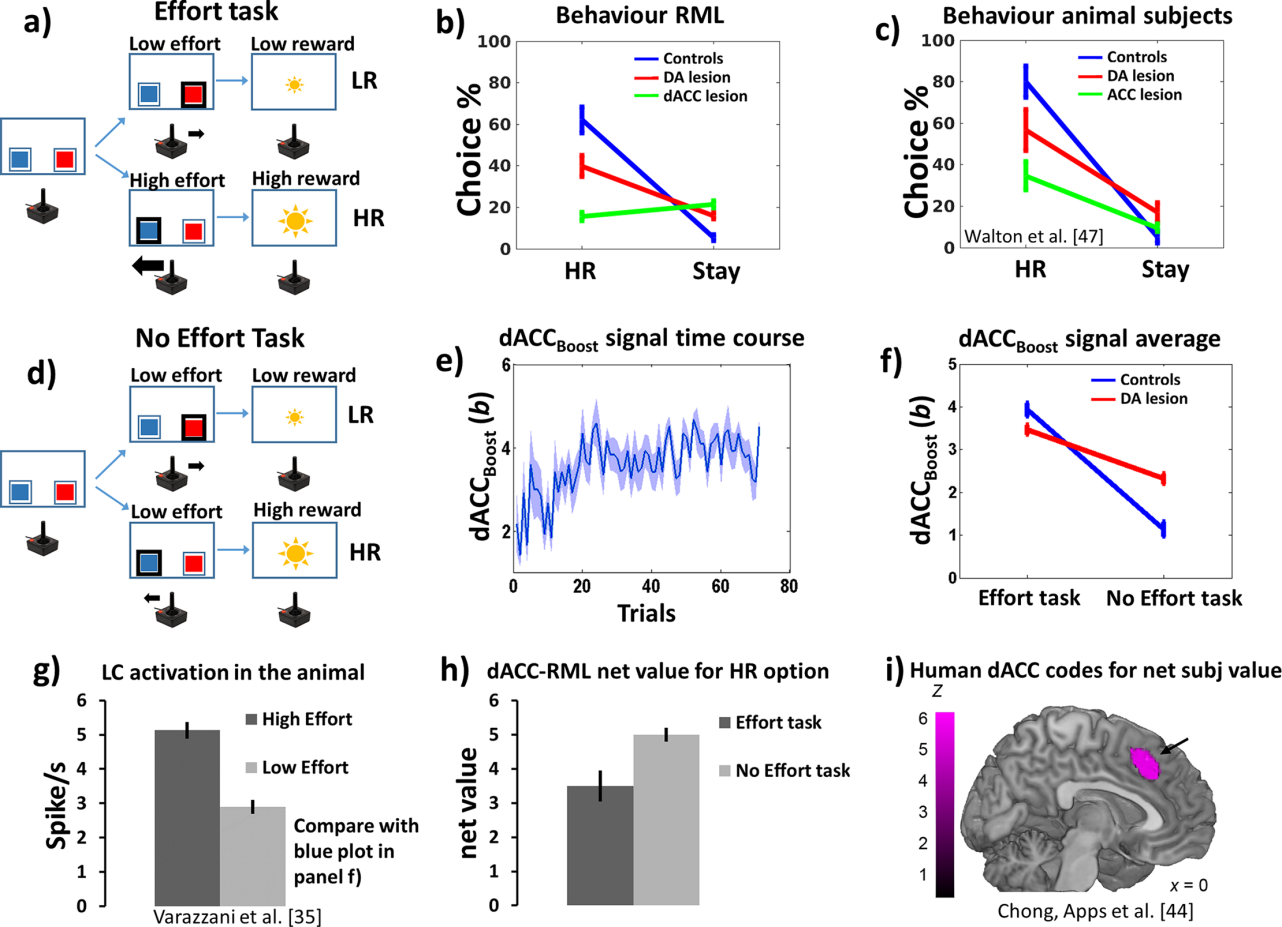
Simulation 2a: Methods and Results. **a)** Effort task, where a high effort choice (thick arrow from joystick) leading to high reward (HR, large sun) was in competition with a low effort choice (thin arrow) leading to low reward (LR, small sun). **b)** Behavioural results (average HR/(LR+HR) ratio ±s.e.m., and average Stay/(LR+HR+Stay) choices ratio percentage ±s.e.m.) from RML and **c)** empirical data from rodents [47], in controls (blue), DA lesioned (red) and ACC lesioned (green) subjects. **d)** No Effort task, same as **a)** but with both options implying a low effort (thin black arrows). **e)** dACC_Boost_ efferent signal (boosting level *b*) time course over trials (average across simulations ± s.e.m.). **f)** dACC_Boost_ efferent signal (*b;* average across time and simulations) as a function of task type (Effort or No Effort task) and DA lesion. The boosting value is higher in the Effort task (main effect of task), but there is also a task x lesion interaction indicating the dACC_Boost_ attempts to compensate the loss of DA in the No Effort task (see main text). Results from the dACC lesion are not reported, as the simulated lesion targeted the dACC itself, leading to an obvious reduction of dACC_Boost_ activity. **g)** LC activity as a function of physical effort in the rhesus monkey [35]. Like in the RML (panel f, blue plot), the LC activity (controlled by the dACC_Boost_) is higher for high effort condition. **h)** RML net subjective value computed in both dACC modules (sum of net values from both dACC modules, Equation S18 in S1 File) for the HR choice as a function of effort. **i)** Like in the human brain [44] the RML dACC computes also the net value (i.e. the value discounted by the expected cost) of choices.

We administered to the RML a 2-armed bandit task with one option requiring high effort to obtain a large reward, and one option requiring low effort to obtain a small reward (here called Effort task [46,47]; Fig 4A). We also administered to the model a task where both options implied a low effort (called No Effort task; Fig 4D). The tasks were also administered to a dACC-lesioned and to a DA-lesioned RML (simulated, respectively, by reducing all neural activations in both the dACC modules and by reducing all VTA outputs; further details about RML simulations and the experimental data from rodents can be found in Simulation 2a in S1 File).

Before the execution of the Effort task, the RML learned the reward values in a task where both options implied low effort (No Effort task). Besides the high effort and low effort choices, the model could choose to execute no action if it evaluated that no action was worth the reward (“Stay” option). Animal data for comparison are from [47] (see Simulation 2a in S1 File).

#### Simulation results and discussion

At the behavioural level (Fig 4B, blue), the RML, like animal subjects [46,47] (Fig 4C, blue), prefers choosing the high-effort-high-reward option (HR) during the Effort task (t(11) = 4.71, p = 0.0042). Again in agreement with rodent data [46,47], both DA and dACC lesions (Fig 4B and 4C, red and green) change this behaviour in a similar manner. Compared with controls, DA lesion increases both the number of choices for low-effort-low-reward (LR) option (t(11) = 3.71, p = 0.0034) and how often the model refuses to engage in the task (“Stay”; t(11) = 18.2, p < 0.0001). dACC lesion leads to the same pattern, with both an increase of LR preference (t(11) = 13.6, p < 0.0001) and of Stay options (t(11) = 11.6, p < 0.0001).

At the neural level, the dACC_Boost_ increased the boosting level (*b*) in the Effort task (Fig 4F; main effect of task, F(1,11) = 231.73, p < 0.0001) enhancing both LC and VTA output. Also nonhuman primates show the same LC effort-related modulation, with a higher LC activation for high effort choices [35] (Fig 4G).

**Fig 5:**
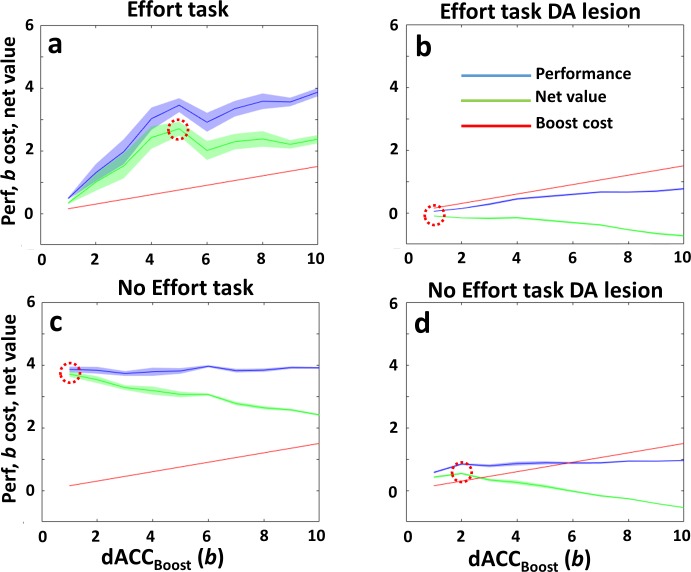
Cost-benefits plots and optimal control of *b* in the dACC_Boost_ module. To obtain these plots we systematically clamped *b* at several values (from 1 to 10, x axis of each plot) and then we administered the same paradigms of Fig 4B and 4D (all the combinations Effort x DA lesion). In all the plots, y axis represents simultaneously performance in terms of average reward signal to dACC_Boost_ (blue plots), boosting cost (red plots) and net value (performance–boost cost, described by Eq 6B). **a)** Effort task, no lesion. Plot showing RML behavioural performance as a function of *b* (blue plot), boosting cost (red plot, Eq 6B in Methods) and net value for the dACC_Boost_ module (green plot, resulting from Eq 6B). Red dotted circles highlight the optimal *b* value which maximizes the final net reward signal received by the dACC_Boost_ module. (maximum of green plot) **b)** Effort task, DA lesion. Same as a), but in this case the RML was DA lesioned. Due to lower average reward signal (blue plot), the net value (green) decreases monotonically, because the cost of boosting (red plot) did not change. Red dotted circle highlights the optimal *b* value, which is lower than in a). It must be considered that, although the optimal *b* value is 1, the average *b* (as shown in figures 5b and s11b) is biased toward higher values, as it is selected by a stochastic process (Eq 4) and values lower than 1 are not possible (asymmetric distribution). **c)** No Effort task, no lesion. In this case, being the task easy, the RML reaches a maximal performance without high values of *b* (blue plot is flat), therefore the optimal *b* value is low also in this case. **d)** No Effort task, DA lesion. As shown also in Fig 4B, in this case the optimal *b* value (dotted circle), is higher than in c), because a certain amount of boosting is necessary to avoid the preference for “Stay” option, which has no costs but also provides no reward. This ensures a minimal behavioural energization to prevent apathy and get a large reward paying a minimal cost (as it is a No Effort task). Plots are average on 40 simulations, error shadows mean s.e.m.

The plot in Fig 4E shows how the RML learns over trials to boost catecholamine release, representing the trial-by-trial optimization process to find the best intensity of modulation over both VTA and LC. Increased *NE* influences decision-making in the dACC_Act_ (effect of *NE* on action cost estimation in decision-making process, Eq 2 in Methods), facilitating effortful actions, while increased DA affects learning in the dACC_Act_ (Eqs 1 and 7A in Methods), increasing the reward signal related to effortful actions. At the same time, boosting catecholamines has a cost (Eq 6B in Methods), so that the higher *b*, the higher was the reward discount for the dACC_Boost_ module. The result of these two opposite forces (maximizing performance by catecholamines boosting and minimizing the cost of boosting itself) converges to the optimal value of *b* and therefore of catecholamines release by VTA and LC (Fig 5A). After DA lesion, the dACC_Boost_ decreased boosting output during the Effort task, while it increased the boosting output during the No Effort task (Fig 4F, red; task x lesion interaction F(1,11) = 249.26, p < 0.0001). Decreased boosting derives from decreased DA signal to dACC_Boost_ module (Fig 5B). Increased boosting *b* in No Effort task can be interpreted as a compensatory mechanism ensuring the minimal catecholamines level to achieve the large reward (HR option) when just a low effort is necessary (Fig 5D). In other words, when the incentive is high (high reward available) and the effort required to obtain the reward is low, the RML predicts that the DA lesioned animal would choose to exert some effort (boosting up the remaining catecholamines) to promote task engagement versus “Stay” option.

Finally, human dACC activity is known to covary not only with effort exertion, but also with net subjective value in effortful tasks (i.e. the expected value of an action discounted by its associated expected effort) [44,54,55]. In Fig 4H, we show how the combined signal from both dACC modules (Equation S18 in S1 File) codes also for the net subjective value, in comparison with human fMRI from [44] (Fig 4I).

### Simulation 2b: Performance recovery after DA lesion, in cost/benefit trade off conditions

In DA lesioned subjects, the preference for HR option can be restored by removing the difference in effort between the two options [47], that is, by removing the critical trade-off between costs and benefits. In Simulation 2b, we show how the RML can recover a preference toward HR options, as demonstrated empirically in experimental paradigms used in rats. We focused specifically on recovery after DA lesion. Our choice was aimed at investigating the consequences of DA lesion at cortical-subcortical level and how these can be modulated by the environment, to open a view on future translational scenarios about DA-related neuropsychiatric disorders. We elaborate on the latter topic in the Experimental Predictions section.

#### Simulation methods

**Fig 6:**
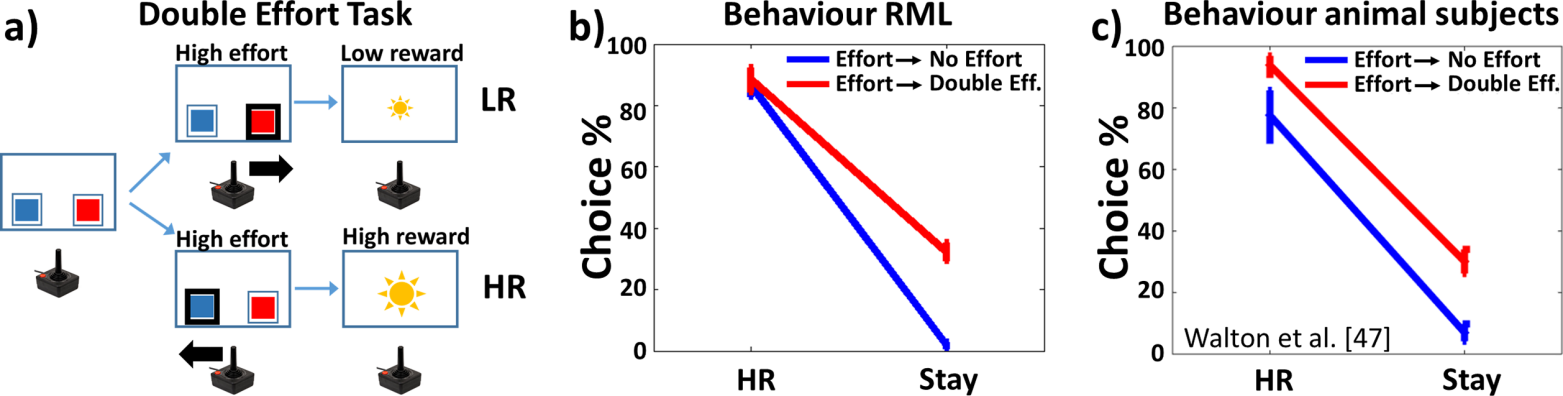
Recovery of HR option preference after DA lesion. **a)** Double Effort task, where both options implied high effort. **b)** Recovery of the preference for HR option (HR/(HR+LR)) when a No Effort task is administered after an Effort task session (Effort → No Effort, blue plot), in both RML and **c)** animals [47] (mean percentage ± s.e.m.). Same phenomenon when a Double Effort session follows an Effort one (Effort → Double Effort, red plot). Note that in this case the number of “Stay” choices (Stay/number of trials) increased, simulating the emergence of apathic behaviour.

The same DA lesioned subjects of Simulation 2a were exposed to either a No Effort task (where both the option required low effort) or a Double Effort task (where both the options required a high effort) (Fig 6A). All other experimental settings were identical to those of Simulation 2a. Animal data for comparison are from [47] (see also Simulation 2b in S1 File).

#### Simulation results and discussion

DA-lesioned RML performance recovers immediately when a No Effort task is administered after the Effort task (Fig 6B, blue), in agreement with animal data ([47]; Fig 6C, blue). This result shows that performance impairment after DA lesion in the model is not due to a learning deficit (although partial learning impairment must occur due to the role of DA in learning), but rather to down-regulation of catecholamines boosting, driven by dACC_Boost_. A task where both options require a low effort does not need a strong behavioural energization, therefore the information about the high reward location is sufficient for an optimal execution.

The same performance recovery occurs also in a task where both options are effortful (Double Effort task, Fig 6B, red), again in agreement with experimental data (Fig 6C, red). Also in this case, when there is no trade-off between costs and benefits (both options are the same in terms of effort), the information about high reward location is sufficient to execute the task optimally, although there is a reduced catecholamine boosting. Nonetheless, differently from the previous scenario, apathy emerges here (percentage of “Stay”). Indeed, the RML often refuse to engage in the task; rather than working hard to get the high reward (whose position is well known) it prefers to remain still. Apathic behaviour in this experiment is more evident than in Fig 4B and 4C, because both RML and animals are forced to make an effort to get a reward, while in Simulation 2a (Fig 4B) they could opt for the low effort-low reward choice.

### Simulation 2c: Adapting cognitive effort in a working memory (WM) task

NE neuromodulation also plays a crucial role in WM, improving signal-to-noise ratio by gain modulation mediated by *α*2-A adrenoceptors [37,56]. A low level of NE transmission leads to WM impairment [57,58]. At the same time, as described above, NE is a major biological marker of effort exertion [35,59]. Besides NE release by the LC, experimental findings showed that also dACC activity increases as a function of effort in WM tasks [19,20,60]. Here we show that the same machinery that allows optimal physical effort exertion (Simulation 2a) may be responsible for optimal catecholamine management to control the activity of other brain areas, thus rooting physical and cognitive effort exertion in a common decision-making mechanism. This is possible because the design of the RML allows easy interfacing with external modules (Fig 1 and Methods).

#### Simulation methods

**Fig 7:**
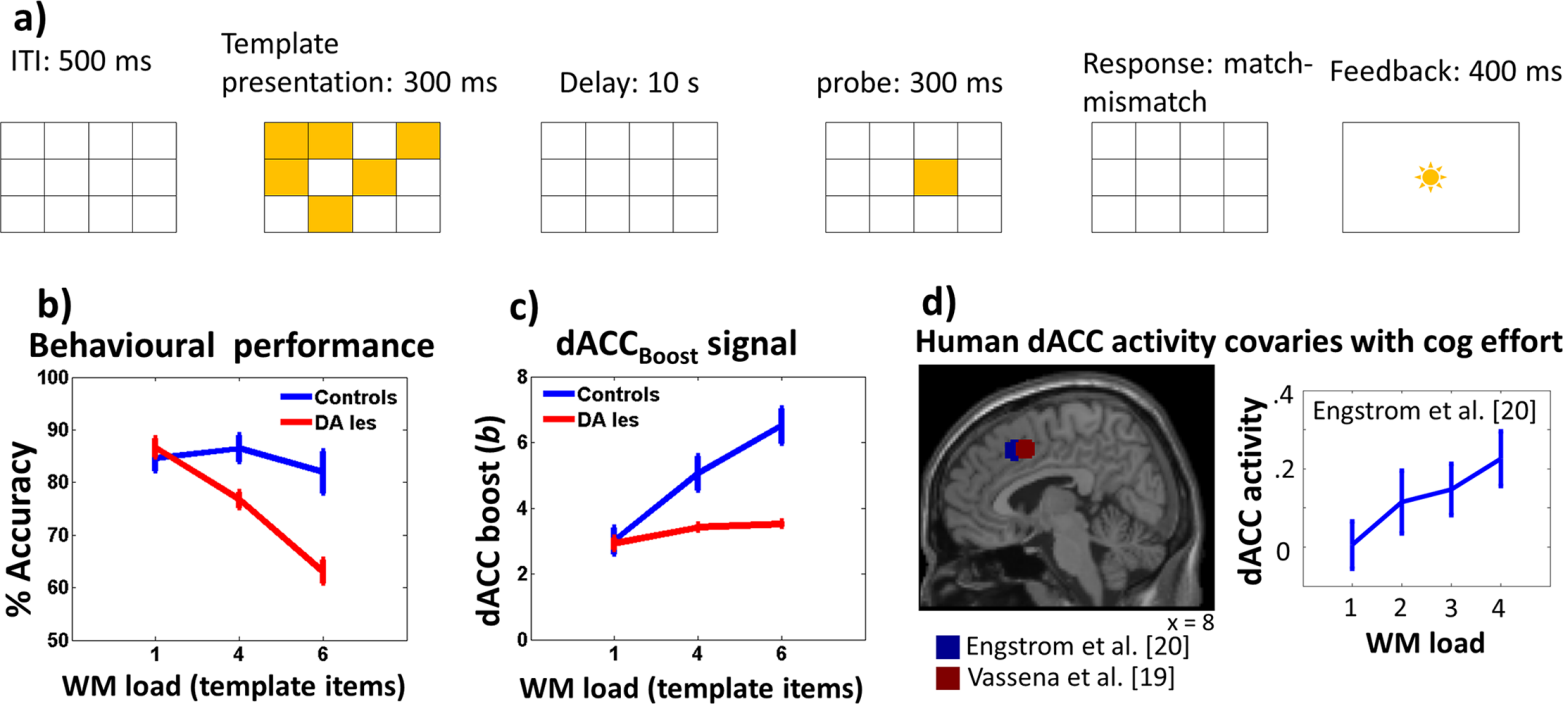
Simulation 2c: Methods and results. **a)** Delayed Matching-to-sample task: events occurring in one trial. **b)** RML behavioural performance as a function of memory load and DA lesion (±s.e.m.). **c)** dACC_Boost_ output as a function of memory load and DA lesion (±s.e.m). **d)** Local maxima in which human dACC activity covaries with cognitive effort (left) and dACC activity as a function of memory load in a WM task ref (right, from blue coordinates).

We connected the RML to a WM model (FROST model; Ashby et al. 2005; see "FROST model description" section in S1 File). Information was exchanged between the two models through the state/action channels in the dACC_Act_ module and the external LC output. The FROST model was chosen for convenience only; no theoretical assumptions prompted us to use this model specifically. FROST is a dynamical recurrent neural network simulating a macro-circuit involving the DLPFC, the parietal cortex and the basal ganglia. This model simulates behavioural and neurophysiological data in several visuo-spatial WM tasks. FROST dynamics simulates the effect of memory loads on information coding, with a decrement of coding precision proportional to memory load (i.e. the number of spatial locations to be maintained in memory). This feature allows to simulate the increment of behavioural errors when memory load increases [61]. In this simulation, the external LC output improves the signal gain in the FROST DLPFC neurons, increasing the coding precision of spatial locations retained in memory (Equation S22 in S1 File), thus improving behavioural performance. We administered to the RML-FROST circuit a delayed matching-to-sample task with different memory loads (a template of 1, 4 or 6 items to be retained; Fig 7A). We used a block design, where we administered three blocks of 70 trials, each with one specific memory load (1, 4, or 6). In 50% of all trials, the probe fell within the template. The statistical analysis was conducted by a repeated measure 3 × 2 ANOVA (memory load by DA lesion).

#### Simulation results and discussion

At the behavioural level, the FROST-RML system maintain a good performance also for high memory loads (Fig 7B, blue plot). At the neural level, the dACC_Boost_ module dynamically modulates catecholamine release as a function of memory load, in order to optimize performance (Fig 7C, blue plot; main effect of memory load on dACC_Boost_ output: F(2,22) = 16.74, p < 0.0001). The computational mechanisms involved in this effect are the same as described in Simulation 2a: The dACC_Boost_ enhances both VTA and LC output, balancing the performance benefits of catecholamines boosting versus the intrinsic cost of boosting. For this reason, when the task is easy (low memory load), catecholamines are low. There is no need to boost in this case: Boosting would be just a cost. In contrast, when the task becomes harder (higher memory loads), catecholamines release increases to keep performance (and reward) high (same mechanism depicted in Fig 5). RML-like dACC activity was found also in healthy humans [19,20] during WM and mental arithmetic tasks (Fig 7D).

In case of DA lesion, at behavioural level, this results in poor performance in particular for high memory loads, when a high level of *NE* is necessary (Fig 7B, red plot; lesion × memory-load interaction: F(2,22) = 8.6, p = 0.0017). This behavioural pattern is due to the consequent disruption of VTA-dACC-LC interaction, leading to a devaluation of boosting and the consequent decision (by the dACC_Boost_ module) of downregulating LC activity (Fig 7C, red plot; main effect of DA lesion on LC output: F(1,11) = 24.88, p < 0.0001). This happened especially for high memory loads (lesion × memory-load interaction: F(2,22) = 7.1, p = 0.0042).

### Simulation 3: Reinforcement learning, meta-learning and higher-order conditioning

Animal behavior in the real world is seldom motivated by conditioned stimuli directly leading to primary rewards. Instead, behavior is guided by higher-order conditioning, bridging the gap between reward and behavior. However, a unifying account explaining behavioral results and underlying neurophysiological dynamics of higher-order conditioning is currently lacking. First, at the behavioral level, literature suggests a sharp distinction between higher-order conditioning in classical versus instrumental paradigms. Indeed, although it is possible to train animals to execute complex chains of actions to obtain a reward (instrumental higher-order conditioning, [62]), it is impossible to install a third- or higher-order level of classical conditioning (i.e. when no action is required to get a reward [63]). Although the discrepancy has been well known for decades, its reason has not been resolved. Second, a number of models have considered how TD signals can support conditioning and learning more generally [64,65]. However, no model addressing DA temporal dynamics also simulated higher-order conditioning at behavioural level.

Here we use the RML to provide a unified theory to account for learning in classical and instrumental conditioning. We show how the RML can closely simulate the DA shifting in classical conditioning (Simulation S2 and Fig F in S2 File). We also describe how the VTA-dACC interaction allows the model to emancipate itself from primary rewards (higher-order conditioning). Finally, we investigate how the synergy between the VTA-dACC_Boost_ and LC-dACC_Boost_ (the catecholamines boosting dynamics) is necessary for obtaining higher-order instrumental conditioning and how this process could be considered one of the foundations of *intrinsic motivation*. This provides a mechanistic theory on why higher-order conditioning is possible only in instrumental and not in classical conditioning.

### Simulation 3a: Higher-order classical conditioning

As VTA can vigorously respond to conditioned stimuli, it is natural to wonder whether a conditioned stimulus can work as a reward itself, allowing to build a chain of progressively higher-order conditioning (i.e. not directly dependent on primary reward). However, for unknown reasons, classical higher-order conditioning is probably impossible to obtain in animal paradigms [63,66]. We thus investigate what happens in the model in such a paradigm.

#### Simulation methods

We first administered a first-order classical conditioning paradigm. We then conditioned a second cue by using the first CS as a non-primary reward. The same procedure was repeated up to third-order conditioning. Each cue was presented for 2s followed by the successive cue or by a primary reward. All cue transitions were deterministic and the reward rate after the third cue was 100%.

#### Simulation results and discussion

**Fig 8:**
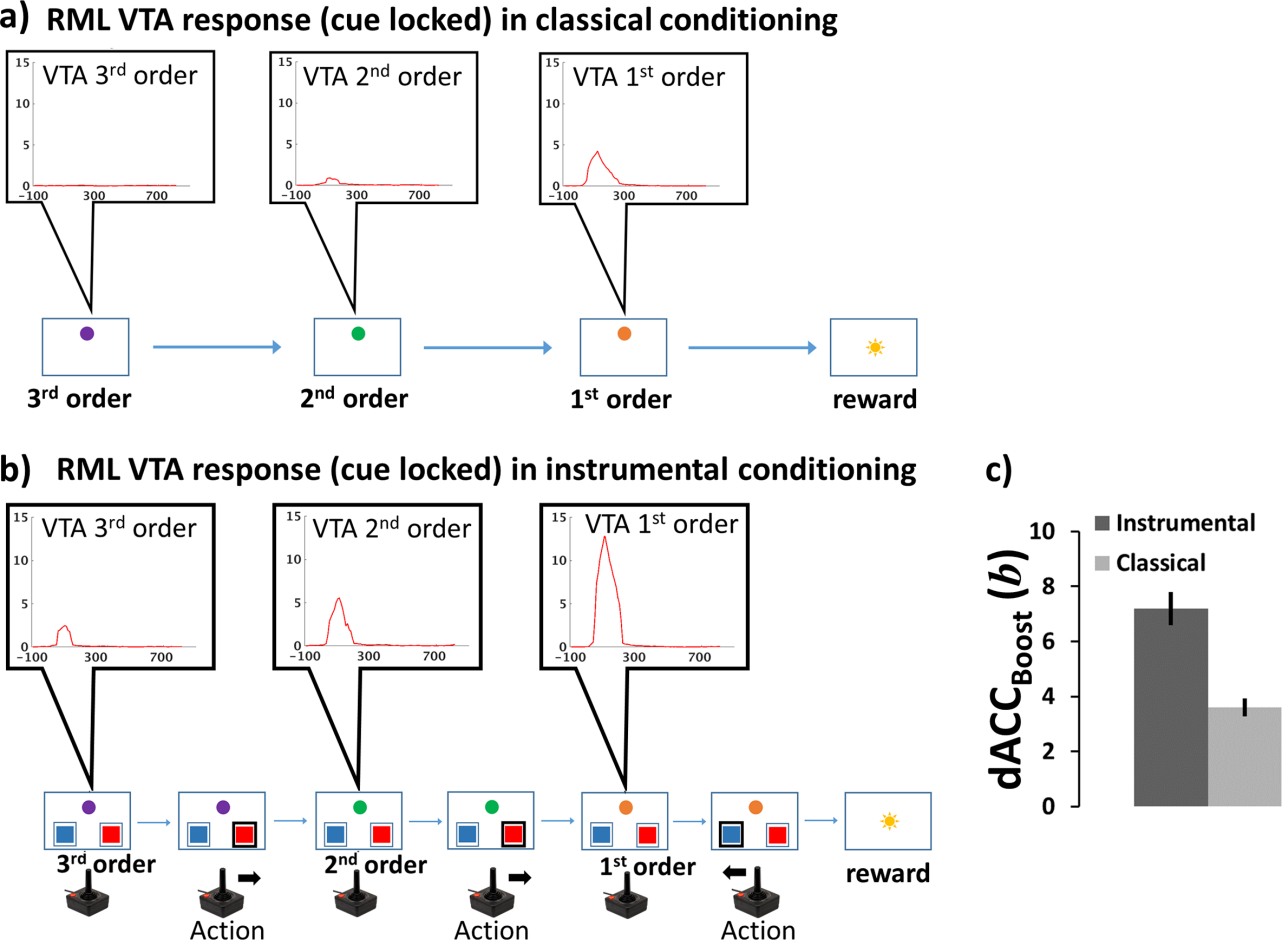
Simulation 3a-b: Methods and results. **a)** Experimental paradigm for higher-order classical conditioning (lower row) and cue-locked VTA response (upper row). The task consisted of a sequence of conditioned stimuli (colored disks) followed by primary reward (sun). Already at the second conditioning order, VTA activity results almost absent. **b)** During a higher-order instrumental conditioning (lower row), the VTA response (upper row) remains sustained up to the third order. **c)** Average dACC_Boost_ efferent signal (*b*± s.e.m.) in classical and instrumental paradigms. In instrumental paradigm the efferent boosting signal is higher, enhancing the VTA activity over different conditioning orders.

In Fig 8A we show the VTA response locked to the onset of each conditioned stimulus. Surprisingly, but in agreement with experimental animal data, the conditioned cue-locked DA release is strongly blunted at the 2^nd^ order, and disappeared almost completely at the 3^rd^ order. This aspect of VTA module dynamics is because, at each order of conditioning, the cue-locked DA signal is computed as the temporal derivative of reward prediction activity from dACC_Action_ (Equation S5b in S1 File). This mechanism implies a steep decay of the conditioning effectiveness of non-primary rewards, because the reinforcing property of cues becomes lower at each order of conditioning. From an ethological viewpoint, it makes sense that the weaker is the link between a cue and a primary reward, the weaker should be its conditioning effectiveness. Nonetheless, as we describe in the following paragraph, this phenomenon is partially counteracted in instrumental conditioning, making higher-order conditioning effective.

### Simulation 3b: Higher-order instrumental conditioning

Differently from classical conditioning paradigms, animal learning studies report that in instrumental conditioning it is possible to train complex action chains using conditioned stimuli (environmental cues) as reward proxies, delivering primary reward only at the end of the task [62].

#### Simulation methods

We administered to the RML a maze-like problem, structured as a series of binary choices before the achievement of a final reward (Figure E in S1 File). Each choice led to an environmental change (encoded by a colored disk, like in Fig 2). The training procedure was the same as for higher-order classical conditioning. We first administered a first-order instrumental conditioning (2-armed bandit task). Then, we used the conditioned environmental cue as non-primary reward to train the RML for second-order conditioning. The procedure was repeated up to third-order conditioning. State-to-state transitions were deterministic and primary reward rate was 100% for correct choices and 0% for wrong choices.

#### Simulation results and discussion

At the end of training, the system was able to perform three sequential choices before getting a final reward, with an average accuracy of 77.3% (90% C.I. = ±13%) for the first choice (furthest away from primary reward; purple disk, Fig 8B); 95.8% (90% C.I. = [4.2, 5.6]%) for the second; and 98% (90% C.I. = ±0.4%) for the third choice (the one potentially leading to primary reward; orange disk, Fig 8B). Fig 8B shows the cue-locked VTA activity during a correct sequence of choices. Differently from classical conditioning, the DA signal amplitude persists over several orders of conditioning, making colored disks (also far away from final reward) effective non-primary rewards, which are able to shape behaviour. It is worth noting that in this simulation the RML self-enhances DA levels to energize behaviour also when primary reward is not available, i.e. it implements intrinsic motivation.

The reason for this difference between classical and instrumental conditioning, is in the role played by the dACC_Boost_ module, and is based on the very same mechanisms underlying optimal control on effort exertion (Simulations 2a-c). Fig 8C compares average boosting levels *b* (efferent signal of dACC_Boost_) in classical and instrumental conditioning. The dACC_Boost_ learned that boosting catecholamines was useful in instrumental conditioning; furthermore it learned that it was not useful in classical conditioning (t(11) = 5.64, p < 0.0001). This decision amplified DA release during task execution only in instrumental conditioning (compare Fig 8A and Fig 8B). Enhanced VTA activity during the presentation of conditioned stimuli (the colored lights indicating a change in the problem space) means more effective higher-order conditioning, therefore a more efficient behaviour. Conversely, in classical conditioning, the model does not need to make any motor decision, as the task consists exclusively of passive observation of incoming cues (colored lights). Therefore, boosting NE and/or DA does not affect performance (reward amount), as this is completely decided by the environment. In this case, boosting would only be a cost (Eq 6B), and the dACC_Boost_ module learned not to boost, with a low DA levels for conditioned stimuli. This explains the strong limitations in establishing higher-order classical conditioning, shows how effort control is involved in higher-order conditioning, and how optimal effort regulation can motivate behaviour also when there is no immediate primary reward available (intrinsic motivation).

## Discussion

We proposed a novel perspective on the neurobiology of decision-making, showing that the recurrent interaction between the dACC and the catecholaminergic brainstem nuclei can generate meta-learning processes, which optimize neural parameters and therefore decision-making in interaction with a wide range of different environments and problems. The RML, the neuro-computational model implementing this novel perspective explains a wide array of heterogeneous empirical findings, including learning rate optimization, effort exertion in physical and cognitive tasks, and higher-order conditioning in classical and instrumental paradigms.

The first meta-learning process we analyzed concerned learning rate (Simulation 1). The RML provides an explicit theory and neuro-computational architecture of how autonomous control of learning rate can emerge from dACC-LC interaction. We propose that the dACC provides RL signals to the LC, about the statistical structure of the environment; in turn, the LC processes those signals to select optimal learning rate by approximating a Bayesian learner. This explains why both structures are necessary for optimal control of flexibility [4,32,33], and why empirical findings indicate that dACC and LC activity are respectively related to RL computation [21] and volatility estimation [30].

The second meta-learning process concerned effort exertion, and optimal allocation of both cognitive and physical effort to achieve a goal (Simulations 2a-c). We proposed that investing (cognitive or physical) effort and controlling associated costs is based on the same computational mechanisms involved in action selection, with one difference: in effort optimization, decision-making is not about actions toward the environment, but concerns the amount of catecholamines that must be released. Moreover, the RML generalizes this mechanism to virtually any cognitive domain, showing how the dACC-brainstem ensemble can work as a provider of optimal control signals (catecholamines) to other brain areas to maximize success while minimizing costs. Finally, effort control is itself modulated by the same mechanisms optimizing learning rate for action selection. This aspect provides near optimal meta-flexibility to cognitive control, a novelty that merges cognitive control with Bayesian learning.

The third meta-learning process that we simulated concerned intrinsic motivation via control over reward signals (both primary and non-primary). Thus, the (primary or nonprimary) reward signal does not depend exclusively on an environmental variable (the reinforcer), but instead can be proactively modulated to increase the value of effortful actions (thus energizing behaviour; Eq 6A, simulations 2a-c) or to increase the value of non-primary rewards (simulations 3a-b). The latter mechanism allowed explaining why higher-order conditioning is possible in instrumental but not in classical paradigms. Moreover, as VTA activity is modulated by the same signal modulating *NE* release (*b* from dACC_Boost_), this feature provides a unified theoretical view on optimal effort allocation and control over motivational and learning aspects.

Although we described them separately, in the RML, learning rate, effort estimation and reward-related processes are integrated and mutually dependent. For example, dynamic control of learning rate (*λ*) is based on RL signals from dACC modules. Learning rate modulation influences both decision-making for action selection and for boosting control (*b*). Boosting control modulates in parallel both LC and VTA, modulating both performance (*NE*) and learning (*DA*). Catecholamine modulation changes behavioural performance, influencing action selection and environmental feedback, thus influencing LC control over learning rate.

### Relationships to other models

#### RL models

The RML belongs to a set of computational models suggesting RL as main function of mammalian dACC [67]. For example, the main idea that dACC is a state-action-outcome predictor is inherited from previous RL neural models (the RVPM and PRO) that already tried to provide a unified view on dACC function (see [3] for a review). The RVPM, in particular, is a subcomponent of the RML model (Model description: dynamical form, in S1 File). This implies that the RML can also simulate the results obtained by the RVPM (e.g., congruency effects, error likelihood estimation), extending even further the amount of empirical data that can be explained by this framework. Although the RML goes beyond these earlier works, by implementing meta-learning and higher-order conditioning, it shares with them the hypothesis that PE plays a core role for learning and decision-making. Indeed, we hypothesize that PE is a ubiquitous computational mechanism, which allows both dACC operations (Eqs 1 and 3) and the approximation of optimal learning rate in the LC (Eq 5A–5D).

#### Hierarchical RL models

Recent computational neuroscience of RL and decision-making focused on hierarchical architectures. For instance, Alexander and Brown [68] proposed a hierarchical RL model (based on their previous PRO model), where hierarchical design is implemented within the dACC, unfolding in parallel with a hierarchical model of the DLPFC. In this model, PE afferents from hierarchically lower dACC layers work as an outcome proxy to train higher layers; at the same time, error predictions formulated at higher layers of DLPFC modulate outcome predictions at lower ones. DLPFC-dACC communication is horizontal (i.e. between layers sharing the same hierarchical level), consisting in PE afferents from dACC to DLPFC, to update predictions. This architecture successfully learned tasks where information is structured at different abstraction levels (like the 1-2AX task), exploring the RL basis of autonomous control of information access to WM.

Also Holroyd and McClure [24] proposed a model exploiting hierarchical RL architecture (the HRL), where the dorsal striatum played a role of action selector, the dACC of task selector and the prelimbic cortex (in rodents) of context selector (where and when to execute a task). Moreover, each hierarchical layer implements a PE-based cognitive control signal that attenuates the costs of action (or task) selection on the lower hierarchical level. This model can explain a wide variety of data about task selection and decision-making in cognitive and physical effort regulation.

The RML differs from these two models for the following reasons. First, it can provide a theoretical account for a broad range of domains (from effort modulation to higher-order conditioning), while having a lower complexity (number of fixed parameters). Second, the RML lacks a genuine hierarchical structure. Its dynamics is emergent from the interaction between cortical and subcortical circuits, allowing meta-learning. This means that the RML provides a recurrent rather than hierarchical theory on the generation of cognitive control signals, without ruling out the relevance of hierarchical mechanisms like those implemented in the HER and HRL.

#### Adaptive effort allocation models

The RML represents cognitive control as dynamic selection of effort exertion, a mechanism that has been recently studied also by Verguts et al. [6], where effort allocation was framed as a decision-making problem. In this model, effort exertion was dynamically optimized by the dACC as a process of RL-based decision-making, so that effort levels were selected to maximize long-term reward. This solution successfully simulated many experimental results from cognitive control and effort investment. The RML makes a step forward, by introducing a mechanism that regulates flexibility of cognitive control itself. Indeed, the interaction between dACC and LC ensures near optimal control of learning rate also in the dACC_Boost_ module. This makes possible to modulate the plasticity of decision-making about effort exertion, while the model is interacting with the environment. Moreover, the RML extends the modeling of cognitive control also to learning and motivation (VTA modulation), describing how LC and VTA influence each other while optimizing behaviour.

A second model by Verguts [69] described how dACC could implement cognitive control by functionally binding two or more brain areas by theta-frequency-locked activation bursts; the theta-wave amplitude would be proportional to the level of control. This theory describes how but not when (and neither how much) control should be exerted. The mechanisms proposed in the RML are complementary to this theory, hypothesizing how, when, and to what extent the dACC itself can decide to modulate theta bursts amplitude.

Le Bouc et al. [70] recently proposed an interesting model-based behavioural analysis on Parkinson disease (PD) patients on and off medication, while executing a physical effort task. Their model aimed at choosing a force exertion level to maximize the expected net value during an effort-based decision-making task. They found that off medication patients had a reduced willingness for exerting effort (apathy) and a slower effort output when this was produced (motor impairment). The authors found that this behavioural pattern was captured by two different free parameters of the model. Apathy was captured by the free parameter coding for reward sensitivity, while motor impairment by the free parameter coding for the rate of motor activation. The RML provided similar results about apathy and reward sensitivity (DA lesion in Simulations 2a-b), with the advantages of ranging its explanatory power across different domains and of being explicitly defined from the neurophysiological point of view, producing in parallel both behavioral and neural dynamics.

In a recent work, the PRO model provided an alternative interpretation of effort-related dACC activation [9], where dACC activation is due to effort intensity prediction (and prediction error) and not to value of exerting effort or to any effort-related control signal. Although this theory is notable for parsimony, it provides no explanation about autonomous control of effort exertion, as it assumes that effort-related effects in the dACC are a byproduct of comparisons between predicted and experienced reward and effort levels. Moreover, it leaves unexplained the causal effects of both DA and dACC lesions and manipulations on effort control itself [47,71].

#### Meta-learning in Bayesian and RL models

Khamassi et al. [72] also hypothesized a role for dACC in meta-learning. The authors proposed a neural model (embodied in a humanoid robotic platform) where the temperature of the action selection process (i.e. the parameter controlling the trade-off between exploration and exploitation) was dynamically regulated as a function of PE signals. Like in the RML, dACC plays both a role in reward-based decision-making and in autonomous control of parameters involved in decision-making itself. Differently from the RML, this model provided a more classical view on PE origin, which were generated by the VTA and not by the dACC like in the RML. Moreover, the mechanism proposed for temperature control was modulated by overall environmental variance (PE), failing to disentangle noise from volatility.

Concerning control of learning rate, earlier *Bayesian models* also adapted their learning rates [4,73,74], proposing a computational account of behavioural adaptation. The main limitations of those models are their loose anatomo-functional characterization, the fact that they are computationally hard (in particular for optimal Bayesian solutions, e.g. [4]), the need for ad hoc forward models of environment statistical structure and the presence of fixed parameters providing the model with explicit information about environmental volatility itself [73,74]. Ad hoc forward models are hierarchically organized, and at the top of this hierarchy, the experimenter defines a priori crucial characteristics (not updatable) about volatility (like the precision of the probability function describing environmental volatility [74]). To the best of our knowledge, the only Bayesian model able to estimate volatility without the need of specifying fixed parameters is the one by Behrens et al. [4], which works only for binary outcomes.

In contrast, the RML provides an explicit neurophysiological theory on how near-optimal control emerges from the dialogue between dACC and brainstem, and it does not rely on fixed parameters providing information about environmental volatility itself. Indeed, we used one hyper-parameter (α in Eq 5C–5D, Methods) representing the minimal assumption that noise variance occurs at higher frequencies than process variance; in other words that environmental changes are slower than fluctuations due to noise. This means that the RML infers environmental volatility in a completely autonomous manner. Moreover, the RML can adapt learning rate in any kind of problem (e.g., binary, continuous), and finally, it integrates approximate Bayesian optimization with other cognitive functions, like effort control and higher-order conditioning.

Interestingly, also Wilson et al. [75] proposed an approximate Bayesian estimator that is based on PE, without the need of specifying a forward model of environmental statistical structure. However, the authors provided a solution for one subclass of volatility estimation problems (the change-point problems) and also in this case, an a priori (fixed) parameter providing information about volatility (the process variance) was needed.

### Experimental predictions

The flexibility of RML, and the explicit neurophysiological hypotheses on which it is based, allow several experimental predictions. In this paper we aimed at presenting the general potential and the theoretical value of the RML, comparing, in a qualitative fashion, the results from our simulations with experimental data from many different domains. A larger use of quantitative approaches to test the experimental predictions derivable from the RML (e.g. model-based data analysis) will be necessary in future work.

Here we list some potential experiments deriving from RML predictions. The first three are sufficiently specific to potentially falsify the model (at least in its neurophysiological interpretation), the others are currently formulated as working hypotheses.

First, the RML architecture suggests that PE signals are generated by the dACC and then converge toward the brainstem nuclei. This hypothesis implies that dACC lesion disrupts DA dynamics in higher-order conditioning, with a consequent impairment in higher-order instrumental conditioning; further, dACC lesion should disrupt LC dynamics related to learning rate control, with a consequent impairment of behavioural flexibility optimization.

A second prediction concerns the mechanisms subtending higher-order conditioning and the difference between classical and instrumental paradigms. In the RML, higher-order conditioning is possible only when the agent plays an active role in learning (i.e., instrumental conditioning). We predict that hijacking the dACC decision of boosting catecholamines (e.g., via optogenetic intervention) would make possible higher-order conditioning in classical conditioning paradigms (ref. simulations 3a-b).

Third, the DA-lesioned RML shows stronger dACC activation during an easy task (without effort) in the presence of a high reward (see Simulation 2a, Fig 4B). This finding can be interpreted as a compensatory phenomenon allowing to avoid apathy (i.e. refusal to engage in the task) if a small effort can make available a big reward. This is an explicit experimental prediction that could be tested both in animal paradigms and in mesolimbic DA impaired humans [76], or in patients with Parkinson’s disease on and off medication [51], therefore providing also possible translational implications.

Fourth, as shown above, the model provides a promising platform for investigating the pathogenesis of several psychiatric disorders. In a previous computational work, we proposed how motivational and decision-making problems in attention-deficit/hyperactivity disorder (ADHD) could originate from disrupted DA signals to the dACC [77]. In the current paper, we also simulated a deficit related to cognitive effort (Simulation 2c) in case of DA deficit. Together, these findings suggest how DA deficit can cause both motivational and cognitive impairment in ADHD, with an explicit prediction on how DA deficit can impair also NE dynamics [78] in ADHD. This prediction could be tested by measuring performance and LC activation during decision-making or working memory tasks, while specifically modulating DA transmission in both patients (via pharmacological manipulation) and RML.

Fifth, another clinical application concerns a recent theory on autism spectrum disorder (ASD) pathogenesis. Recent studies [79,80] proposed that a substantial number of ASD symptoms could be explained by dysfunctional control of learning rate and overestimation of environment volatility. This qualitative hypothesis could be easily implemented and explored quantitatively by altering meta-learning mechanisms in the RML leading to chronically high learning rate and LC activation.

### Limitations

The RML framework has three main limitations. First, in the RML DA plays a role only in learning. As with any other neuromodulator, experimental results suggest a less clear-cut picture, with DA being involved also in performance directly (e.g. attention and WM via DLPFC modulation) [39,81–83]. The goal of our simplified characterization of DA function was to elucidate how the two neuromodulators can influence each other for learning (DA) and performance (NE). Moreover, other theories stress the importance of direct (and hierarchically organized) interaction between the medial prefrontal cortex and the DLPFC in cognitive control [84] and WM function [68]. From this perspective, reduced DA signal to the dACC could directly disrupt the dACC-DLPFC interaction, impairing cognitive control and WM without the involvement of the NE modulation. dACC-DLPFC interaction is a neglected aspect in our model that should be investigated in future works (see next section).

The second limitation is the separation of the LC functions of learning rate modulation (*λ*) and cognitive control exertion. The cost of this separation between these two functions is outweighed by stable approximate optimal control of learning rate and catecholamines boosting policy. It must be stressed that the ACC_Boost_ module receives the LC signal *λ* related to learning rate in any case, making the boosting policy adaptive to environmental changes.

Third, the RML reacts to environmental changes by learning rate modulation, while human and nonhuman primates can use specific events that occurred (episodic control [85]), to trigger policy change for adapting to novel situations. There is also converging evidence that primate dACC (and most likely its homologous area in rats) is critical to perform this type of higher-order inference (see [7] for a short review), and that LC bursts could work as circuit breakers to reset ongoing neural representations and trigger behavioural adaptation driven by episodic control [86]. The lack of contribution by episodic knowledge in behavioural optimization is clearly a limitation of our model, especially if we consider that episodic control can also optimize motivational signals to modulate cognitive effort [84]. We believe that these two adaptive processes (i.e. learning rate control and episodic control) are complementary and run in parallel and that their integration (a possibly arbitration on influencing behaviour) should receive future theoretical investigation.

### Future perspectives

The RML shows how meta-learning involving three interconnected neuro-cognitive domains can account for the flexibility of the mammalian brain. However, our model is not meant to cover all aspects of meta-learning. Many other decision-making dimensions may be optimized by meta-learned too. One obvious candidate is the stochasticity (temperature) of the decision process [87], which arbitrates the exploration/exploitation trade-off. We recently proposed that this parameter is similarly meta-learned trading off effort costs versus rewards [6]. It must be noted that experimental findings indicated a link between LC activation and the arbitration on exploration/exploitation trade-off [88,89], suggesting that the same mechanism used for learning rate optimization could be extended also to this domain. Other aspects from the classical RL modeling framework include discounting rate or eligibility traces [90]; future work should investigate the computational and biological underpinnings of their optimization. Moreover, considering the strong empirical evidence attributing to the dACC a prominent role in foraging (e.g. [91]), future work should focus on how the RML can also face this class of problems, where it is studied not only how mammals optimize choices within a task, but also how they decide when it is convenient to switch to another task, to maximize reward in the long run.

Given the exceptionally extended dACC connectivity [12], other brain areas are likely relevant for the implementation of decision making in more complex settings. For example, we only considered model-free dynamics in RL and decision-making. However, both humans and nonhuman animals can rely also on complex environment models to improve learning and decision making (e.g. spatial maps for navigation or declarative rules about environment features). In this respect, future work should particularly focus on dACC-DLPFC-hippocampus interactions [92,93], in order to investigate how environment models can modulate reward expectations, how the nervous system can represent and learn decision tree navigation [94] and how reward expectations can modulate goal-directed DLPFC representations [84].

Another anatomo-functional aspect that could be investigated concerns the anatomical segregation of the twofold dACC function we described here (dACC_Act_ and dACC_Boost_). Although we remain agnostic about this question, it would be interesting to investigate whether the neural units performing these two types of decision-making operations are overlapping, intermixed, or even segregated in different dACC sectors.

Finally, the RML can work in continuous time and in the presence of noise. These features are crucial to make a model survive outside the simplified environment of trial-level simulations, and allow simulating behaviour in the real world, like, for example, in robotic platforms. RML embodiment into robotic platforms could be useful for both neuroscience and robotics. Indeed, testing our model outside the simplified environment of computer simulations could reveal model weaknesses that are otherwise hidden. Moreover, closing the loop between decision-making, body and environment [95] is important to have a complete theory on the biological and computational basis of decision-making. At the same time, the RML could suggest new perspectives on natural-like flexibility in machine learning, helping, for example, in optimizing plasticity as a function of environmental changes.

## Methods

### Model description

RML architecture was implemented in two versions: a discrete model (simulating inter-trial dynamics) and a dynamical model (a dynamical system simulating also intra-trial dynamics). Both implementations share the same architecture displayed in Fig 1, and follow the same computational principles. All the results reported above were obtained with the dynamical model. Here we introduce the mathematical form of the discrete model, which provides a clearer and more compact RML description. All the simulations (with exception of Simulation 2c, which requires intra-trial dynamics) were replicated with the discrete model (Figures S9-S12 in S2 File), demonstrating that the computational principles founding the RML are independent from specific implementations. We used a single set of parameters across all simulations both for the discrete model (Table 1) and for the dynamical model (Table A in S1 File). Parameters were hand-tuned to ensure acceptable performance in a simple 2-armed bandit task and second-order conditioning task.

**Table 1:** Parameters list and values for discrete model.

Parameter	Value	Meaning	Equation
*ρ*	0.2	TD-learning signal decay	6a
*μ*	0.1	DA dynamics	6a
*τ*	0.6	Softmax temperature	2
*α*	0.3	Kalman filtering meta-parameter	5c-d
*β*	0.2	Learning rate lower bound	5a
*ω*	0.15	Boosting cost	6b

The mathematical description of the dynamical model can be found in the S1 File.

**Fig 9:**
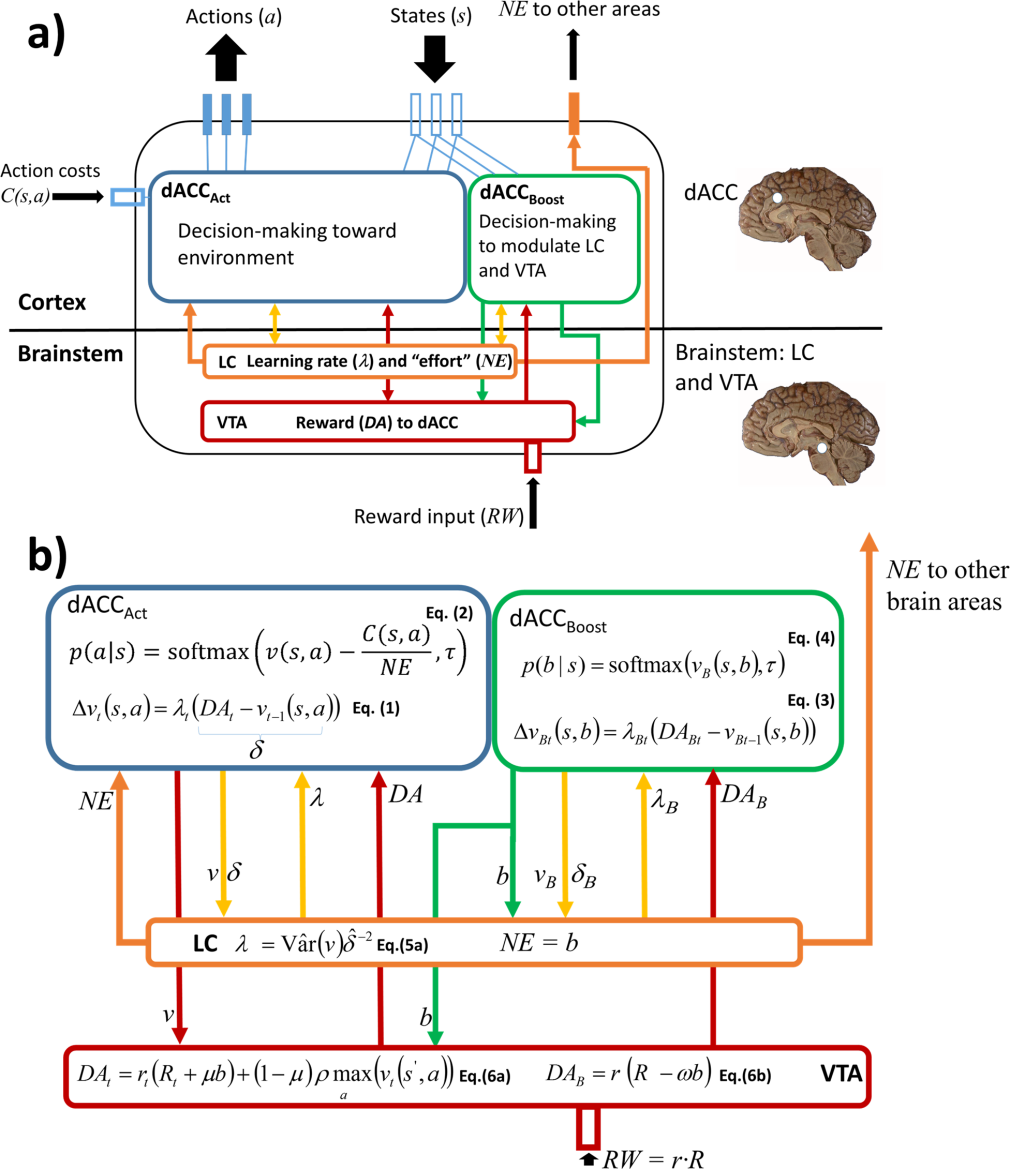
RML overview with equations. **a)** The RML-environment interaction happens through nine channels of information exchange (black arrows) (input = empty bars; output = filled bars). The input channels consist of one channel encoding action costs (*C*), three channels encoding environmental states (*s*), and one channel encoding primary rewards (*RW*). The output consists of three channels coding each for one specific action (*a*), plus one channel conveying LC signals to other brain areas (*NE*). The entire model is composed of four reciprocally connected modules (each in a different color). The upper modules (blue and green) simulate the dACC, while the lower modules (red and orange) simulate the brainstem catecholamine nuclei (VTA and LC). dACC_Act_ selects actions directed toward the environment and learns through first and higher-order conditioning, while dACC_Boost_ modulates catecholamine nuclei output. The VTA module provides *DA* training signals to both dACC modules. The LC controls learning rate (*λ*; yellow bidirectional arrow) in both dACC modules, and effort exertion (promoting effortful actions) in the dACC_Act_ module (orange arrow), influencing their decisions. Finally, the LC signal controlling effort in the dACC_Act_ can be directed also toward other cognitive modules for neuro-modulation. **b)** Model overview with equations embedded. The equations are reported in their discrete form. Communication between modules is represented by arrows, with corresponding variables near each arrow. Variables δ and δ_*B*_ represent the prediction errors from respectively Eqs 1 and 3.

We designed the model such that communication with the external environment is based on 9 channels (Fig 9A). Six channels represent environmental states (*s*) and RML actions (*a*) (3 states and 3 actions). The first two actions are aimed at changing the environmental state (e.g. turning right or left), while the 3^rd^ action means “Stay”, i.e. refusing to engage in the task. There are two other input channels, one dedicated to reward from environment (*RW*) and the other to signal costs of motor actions (*C*). Finally, there is one output channel conveying norepinephrine (*NE*) signals to other brain areas. The RML is scalable by design, i.e. there is no theoretical limit to the number of state/action channels, and neither the number of parameters nor their values changes as a function of task type/complexity.

#### dACC_Act_

The dACC_Act_ module consists in a Q-learning algorithm augmented by meta-learning functions (Fig 9A and 9B, blue box). Here we refer to the performance monitoring part of the dACC_Act_ module as “Critic”, while to the action selection part as “Actor”. The Critic is a performance evaluator and computes reward expectation and PE for either primary or non-primary rewards (higher-order conditioning), learning to associate stimuli and actions to environmental outcomes. The Actor selects motor actions (based on Critic expectation) to maximize long-term reward.

The central equation in this module governs Critic state/action value updates:

$$\Delta v_{t}\left(s,a\right)=\lambda_{t}\left(DA_{t}-v_{t-1}\left(s,a\right)\right)$$ (1)

where *v*(*s*,*a*) indicates the value (outcome prediction) of a specific action *a* given a state *s*. Eq 1 ensures that *v* comes to resemble the environmental outcome encoded by dopaminergic signal (*DA*), which is generated by the VTA module (Fig 9B; Eq 6). It entails that the update of *v* at trial *t* is based on the difference between prediction (*v*) and outcome (*DA*), which defines the concept of PE. The latter is weighted by learning rate *λ* (called also step-size parameter in RL terminology), making the update more (high *λ*) or less (low *λ*) dependent on recent events. We propose that *λ* itself is modulated by the LC based on *v* and PE signals from the dACC_Act_ (Eq 5A).

The *DA* signal, afferent from the VTA, conveys either primary or non-primary reward (higher-order conditioning) and is modulated by the dACC_Boost_ module via parameter *b* (Eq 6A). It is worth noting that the rate of value changing described in Eq 1 depends obviously on *λ*, but also on PE. The latter depends on *DA* magnitude, and thence on the modulation that dACC_Boost_ exerts over the VTA module. For this reason, we can say that the overall rate of learning (i.e. *Δv*) depends on both NE (controlling *λ*) and DA (determining PE) modulations.

Action *a* is selected by the Actor subsystem, which implements action selection (by softmax selection function, with temperature τ) based on state/action values discounted by state/action costs *C*:

$$p\left(a|s\right)=\mathrm{softmax}\left(v\left(s,a\right)-\frac{C\left(s,a\right)}{NE},\tau \right)$$ (2)

where we define softmax(*x*_*i*_,*τ*) = exp(*x*_*i*_/*τ*)/∑exp(*x*_*i*_/*τ*). Function *C* assigns a cost to each state/action couple, for example energy depletion consequent to climbing an obstacle. *C* is modulated by norepinephrine afferents from LC (*NE*), which is itself controlled by the dACC_Boost_ module, via parameter *b* (cf. also Holroyd and McClure, 2015) *NE* levels discount *C*, lowering the perceived costs and energizing behaviour. We remind the reader that the RML can choose not to engage in the task (“Stay”); this option has *C* = 0. In this way, a high level of *NE* energizes behaviour, promoting both high cost actions and reducing the probability that the RML chooses to “Stay”.

The dynamical form of these equations is described in the *dACC*_*Act*_*-VTA system* paragraph in S1 File.

#### dACC_Boost_

The dACC_Boost_ module is an Actor-Critic system that learns only from primary rewards (Fig 9, green box). This module controls the parameters for cost and reward signals in Eqs 1 and 2 (dACC_Act_), via modulation of VTA and LC activity (boosting catecholamines). In other words, whereas the dACC_Act_ decides on actions toward the external environment, the dACC_Boost_ decides on actions toward the internal environment: It modulates brainstem nuclei (VTA and LC), given a specific environmental state. This is implemented by selecting the modulatory signal *b* (*boost signal*), by RL-based decision-making. In our model, *b* is a discrete signal that can assume ten different values (integers 1–10), each corresponding to one action selectable by the dACC_Boost_. The Critic submodule inside the dACC_Boost_ updates the boost values *v*_*B*_(*s*, *b*), via the equation:

$$\Delta v_{B,t}\left(s,b\right)=\lambda_{B,t}\left(DA_{B,t}-v_{B,t-1}\left(s,b\right)\right)$$ (3)

Eq 3 represents the value update of boosting level *b* in the environmental state *s*. The dACC_Boost_ module receives dopaminergic outcome signals (*DA*_*B*_) from the VTA module. As described in Eq 6B, *DA*_*B*_ represent the reward signal discounted by the cost of boosting catecholamines [5,96,97]. Also in Eq 3 there is a dynamic learning rate (*λ*_*B*_), estimated by Eq 5A in the LC. The Actor submodule selects boosting actions based on expected values *v*_*B*_ and temperature *τ*:

$$p\left(b|s\right)=\mathrm{softmax}\left(v_{B}\left(s,b\right),\tau \right)$$ (4)

Referring to Eq 1, the dACC_Boost_ modulates the reward signal by changing the *DA* signal coded in VTA (Eq 6A). Furthermore, dACC_Boost_ also modulates the cost signal by changing parameter *NE* (via LC module, see paragraph below) in the function representing action cost *C* (Eq 2; represented in the Actor within the dACC_Act_). The dynamical form of these equations is described in the *dACC*_*Boost*_*-LC-VTA system* paragraph in S1 File.

#### LC: Control over effort exertion and behavioural activation

The LC module plays a double role (Fig 9, orange box). First it controls cost via parameter *Ne*, as a function of boosting value *b* selected by the dACC_Boost_ module. For sake of simplicity, we assumed *NE* = *b*; any monotonic function would have played a similar role. The *NE* signal is also directed toward external brain areas as a performance modulation signal (Fig 1A; Simulation 2c).

#### LC: Control over learning rate

The LC module also optimizes learning rate in the two dACC modules (∠ and *λ*_*B*_). Approximate optimization of *λ* solves the trade-off between stability and plasticity, increasing learning speed when the environment changes and lowering it when the environment is simply noisy. In this way, the RML updates its knowledge when needed (plasticity), protecting it from random fluctuations. This function is performed by means of recurrent connections between the dACC (both modules) and the LC module, which controls learning rate based on the signals afferent from the dACC. The resulting algorithm approximates Kalman filtering [73,98], which is a recursive Bayesian estimator. In its simplest formulation, Kalman filter computes expectations (posteriors) from current estimates (priors) plus PE weighted by an adaptive learning rate (called Kalman gain). If we define process variance as the outcome variance due to volatility of the environment, Kalman filter computes the Kalman gain as the ratio between process variance and total variance (i.e. the sum of process and noise variance). From the Bayesian perspective, the Kalman gain reflects the confidence about priors, so that high values reflect low confidence in priors and more influence of evidence on posteriors estimation.

The main limitation of this and similar methods is that one must know a priori the model describing the environment statistical properties (noise and process variance). This information is typically inaccessible by biological or artificial agents, which perceive only the current state and outcome signals from the environment. Our LC module bypasses this problem by an approximation based on the information afferent from the dACC, without knowing a priori neither process nor noise variance. To do that, the LC modulates *λ* (or *λ*_*B*_) as a function of the ratio between the estimated variance of state/action-value $\left(V\hat{a}r(v)\right)$ over the estimated squared PE (*δ*^2^):

$$\lambda_{t}=\frac{V\hat{a}r{\left(v\right)}_{t}}{{\hat{\delta }}_{t}^{2}}$$ (5A)

with *β ≤ λ ≤* 1 (*β* is a free parameter indicating the minimal learning rate), to ensure numerical stability.

The process variance is given by:

$$V\hat{a}r\left(v\right)={\left(v_{t}-{\hat{v}}_{t-1}\right)}^{2}$$ (5B)

where $\hat{v}$ is the estimate of *v*, obtained by low-pass filtering tuned by meta-parameter *α*:

$${\hat{v}}_{t}={\hat{v}}_{t-1}+\alpha \left(v_{t}-{\hat{v}}_{t-1}\right)$$ (5C)

The same low-pass filter is applied to the PE signal (*δ*) to obtain a running estimation of total variance *δ*^2^, which corresponds to the squared estimate of unsigned PE:

$${\hat{\delta }}_{t}={\hat{\delta }}_{t-1}+\alpha \left(\left|\delta_{t}\right|-{\hat{\delta }}_{t-1}\right)$$ (5D)

In summary, in Eqs 5A–5D Kalman gain is approximated using 3 components: reward expectation (*v*), PE signals (*δ*) (both afferent from the dACC modules) and a meta-parameter (*α*), defining the low-pass filter to estimate process and total variance. The meta-parameter *α* represents the minimal assumption that noise-related variability occurs at a faster time scale than volatility-related variability. Eqs 5A–5D are implemented independently for each of the two dACC modules, so that each Critic interacts with the LC to modulate its own learning rate. The dACC modules and the LC play complementary roles in controlling *λ*: The dACC modules provide the LC with the time course of expectations and PEs occurring during a task, while the LC integrates them to compute Eq 5A.

The dynamical form of these equations is described in the *dACC-LC system* paragraph in S1 File.

#### VTA

The VTA provides training signal *DA* to both dACC modules, either for action selection directed toward the environment (by dACC_Act_) or for boosting-level selection (by dACC_Boost_) directed to the brainstem catecholamine nuclei (Fig 9, red box). The VTA module also learns to link dopamine signals to arbitrary environmental stimuli (non-primary rewards) to allow higher-order conditioning. We hypothesize that this mechanism is based on DA shifting from primary reward onset to conditioned stimulus (*s*, *a*, or both) onset [99].

$$DA_{t}=r_{t}\left(R_{r}+\mu b\right)+b\left(1-\mu \right)\rho \max_{a}\left(v_{t}\left(s^{\prime },a\right)\right)$$ (6A)

Eq 6A represents the modulated (by *b*) reward signal. Here, *r* is a binary variable indicating the presence of reward signal, and *R* is a real number variable indicating reward magnitude. Parameter *ρ* is the TD discount factor, while parameter *μ* is a scaling factor distributing the modulation *b* between primary (first term of the equation) and non-primary (second term) reward. It is worth noting that when *μ* = 0, Eq 6A simplifies to a Q-learning reward signal.

The VTA signal directed toward the dACC_Boost_ is described by the following equation:

$$DA_{B,t}=r_{t}\left(R_{t}-\omega b\right)$$ (6B)

where *ω* is a parameter defining the cost of catecholamine boosting [5,96,97]. In summary, boosting up DA by *b* (Eq 6A), can improve behavioural performance (as shown in simulations below) but it also represents a cost (Eq 6B). The dACC_Boost_ module finds the optimal solution for this trade-off, choosing the optimal DA level to maximize performance while minimizing costs (for a formal analysis about this optimization process we refer to Verguts et al., 2015). The dynamical form of these equations is described in the *dACC*_*Act*_*-VTA* and *dACC*_*Boost*_*-VTA* paragraphs in S1 File.

#### Control over other brain areas

Finally, the RML can optimize performance of other brain areas via its plug-in loop. It does so via the LC-based control signal (*NE*), which is the same signal that modulates effort (Eq 2; Fig 1A). Indeed, the Actor-Critic function of the dACC_Act_ module is domain-independent (i.e. the state/action channels can come from any brain area outside dACC), and this allows a dialogue with other areas. Moreover, because optimization of any brain area improves behavioural performance, the dACC_Boost_ can modulate (via LC signals) any cortical area to improve performance (see Simulation 2c).

## Supporting information

S1 File Supplementary methods. (PDF)

S2 File Supplementary results. (PDF)
