# Supplementary material for: Dorsal anterior cingulate-brainstem ensemble as a reinforcement meta-learner
Source: PLoS Comput Biol. 2018 Aug 24;14(8):e1006370. doi: 10.1371/journal.pcbi.1006370 (PMC6126878; doi:10.1371/journal.pcbi.1006370)
Supplement: S1 File — (PDF) [file pcbi.1006370.s001.pdf]

Supplementary Methods

Model Description: Dynamical Form.

The RML general schema in its dynamical form is the same as the one described in Figure 1 in the main text. In Table A are reported the parameters for this version of the RML.

Table A. Parameter values for the dynamical version of the RML

Parameter	Value	Meaning	Equation
θ	0.1	Noise filter cut-off (to be used in case of noisy neurons)	S1
ε	0.1	Synaptic weights decay	S1b
γ	0.1	Neural activity decay	S2-4,
σ	0.05	Neural units noise variance	S2-4
μ	0.35	DA dynamics	S5a,b
τ	0.6	Softmax temperature	S7
α	0.0015	Approximate Kalman filtering meta-parameter	S9-10, 13
β	0.055	Learning rate scaling	S14
ω	0.3	Boosting cost	S15

Here follows a detailed description of the RML dynamical implementation. We parceled the description in paragraphs describing the interaction between modules pairs (here called systems).

The dACC$_{Act}$-VTA system. The dACC$_{Act}$ (action selection) - VTA (dopamine) system is shown in Fig S1. Its aim is to learn state-action values from both primary reward and reward signals from the onset of conditioned stimuli (higher order conditioning). The dACC$_{Act}$ module exchanges information with the external environment by means of six channels. Three of them code for environmental states (empty blue bars) and the other three code for actions. The information about states and actions is encoded in the vector **o**, where the first three dimensions (i) represent only states, while the others encode state-action pairs. The activations of the state-action pairs are computed by Kronecker product of state (**s**) and action (**a**) channels ($\mathbf{o} = \mathbf{s} \otimes \mathbf{a}$, $\forall\ i > 3$). Any time a specific environmental state occurs, the corresponding state channel is set to one. If any action is required, the action channels are pre-activated (all set to small number > 0), otherwise, they are set to zero. Vector **o** is connected to the three Critic sub-modules (neuronal triplets) by matrix **W**. Each Critic sub-module computes reward prediction (v units) and prediction error (δ units) related to **o** activation. Each element of the connection matrix **W** (between the input layer **o** and the set of Critic systems) is updated by:

$$\dot{w}_{ji} = o_i \lambda_j \left(\delta_j^{\,+} - \delta_j^{\,-} \right) \qquad \text{S1}$$

where the index j indicates the Critic sub-module (1 to 3 in our implementation, Figure A), δ^+ and δ^- respectively positive and negative prediction error units activation (Equations S3-4), and λ is the dynamic learning rate, controlled by the interaction between the j-th Critic sub-module and the j-th sub-module of the LC (Equation S19). If δ units are noisy (or their baseline activity is greater than zero) their contribution to S1 must be first filtered by a threshold θ, to cut-off spurious contribution by baseline activity. As we used noisy neural units in the Critic, we set $\theta = 0.1$. The connections matrix **W** stores the long-term values of states and state-action couples. To prevent the same state-

action couple to be represented in multiple critic sub-modules (due to reward signal propagation through dACC-VTA serial connectivity, Equation S5b), we introduced a synaptic competition between sub-modules, such as:

$$\dot{w}_{ji} = -\varepsilon w_{ji} \mid j \neq \arg\max_{j}\left(w_{ji}\right) \qquad \text{S1b}$$

where $\varepsilon = 0.1$ is a synaptic decay parameter. The instant value associated to state-action combinations is computed in each value unit v_j inside each Critic sub-module (indexed with j). The v units are noisy leaky integrators. Their update equation is:

$$\dot{\mathbf{v}} = \gamma\left(\mathbf{W}\cdot\mathbf{o}\right) - \gamma\mathbf{v} + \eta \qquad \text{S2}$$

where **v** is the vector of v units, η is a random variable representing noise ($\eta \sim N(0,\sigma)$) and $\gamma = 0.1$ is a decay parameter. The prediction error units (δ) update equations for the j-th Critic sub-module are:

$$\dot{\delta}_j^{\,+} = \gamma\left[DA_j - Tv_j\right]^+ - \gamma\delta_j^{\,+} + \eta \qquad \text{S3}$$

$$\dot{\delta}_j^{\,-} = \gamma\left[Tv_j - DA_j\right]^+ - \gamma\delta_j^{\,-} + \eta \qquad \text{S4}$$

respectively for positive and negative prediction error units. Here $[x]^+$ indicates $\max(0, x)$, while DA is the outcome signal (dopamine) afferent from the VTA module (Equation S5a,b). Finally, T is

the timing signal indicating the expected occurrence of any feedback (for any *j*) in time (boxcar signal with unit amplitude). Here, for simplicity, we set *T* by hand, as a function of any event playing the role of environmental feedback. Alternatively, *T* can be learned trial-by-trial [1]. The outcome signal (*DA*) provided to the *j*-th Critic submodule by the VTA is defined by:

$$DA_j = r(R + \mu b) \mid j = 1 \qquad \text{S5a}$$

$$DA_j = b\left[(1-\mu)\left[\dot{v}_{j-1}\right]^+ + \mu\left(\left[\dot{\delta}^+_{j-1}\right]^+ - \left[\dot{\delta}^-_{j-1}\right]^+\right)\right]^+ \mid j > 1 \qquad \text{S5b}$$

where *r* is a binary signal indicating the presence of a reward (boxcar function with unity amplitude), *R* indicates reward magnitude ($R \neq 0$), and *b* is the boosting signal from the $dACC_{Boost}$ module. In both equations S5a and S5b, $\mu = 0.35$ is a scaling parameter. The time derivatives of the neural units from the *j*-th sub-module are computed as discrete time differences. In all our simulations, we used a time step of 10 network cycles, corresponding to 100 ms, simulating a transient VTA neural response when a prolonged stimulus (*v* and δ) is applied. Rectifications prevent negative neural input when derivatives are negative. As specified in the main text, the VTA signal that allows higher-order conditioning derives from the *v* units input (i.e. DA response locked to conditioned stimulus). Recent single unit data showed a wide range of neural response during conditioning, with many neurons showing response shifting from reward to CS (like in the TD algorithm), while others being influenced by either reward expectation or PE [2], or even coding for primary rewards (Equation S5a) [3]. The terms in equations 5a-b, related to reward expectation $[\dot{v}]^+$ and PE $[\dot{\delta}]^+$ model how the contribution of afferents from the dACC can be combined to generate a prototypical VTA neuron activity shifting from reward to CS onset.

The Actor module, which selects actions based on the Critic's evaluation, is described by the following two equations.

$$h_i = \left(\sum_j w_{ij}\right) - \frac{C_i}{NE} \quad \text{S6}$$

$$p(i \mid \mathbf{h}) = \text{softmax}(h_i, \tau) \quad \forall i \mid 0 < o_i < 1 \quad \text{S7}$$

In Equation S6, C_i is the cost associated to the *i*-th state-action couple (vector **o**), while *NE* is the LC module output (norepinephrine) which is determined by the $dACC_{Boost}$ module. Here we chose $NE = b$, as we here consider the simplest situation in which the LC works like a broadcasting system of the dACC boosting decision. Equation S7 expresses the probability of selecting the action associated to the *i*-th state-action couple (with $\tau = 0.6$ as a temperature parameter) when the *i*-th action was pre-activated (if there is no premotor activation, the $dACC_{Act}$ module emits no action). Like in the main text, here we define $\text{softmax}(x_i, \tau) = \exp(x_i / \tau) / \sum \exp(x_i / \tau)$. Once one action is selected, the corresponding unit is set to one, while all the others having a smaller activation are set to zero. The selected state-action unit remains active until feedback from environment (either primary reward or state transition) is provided.

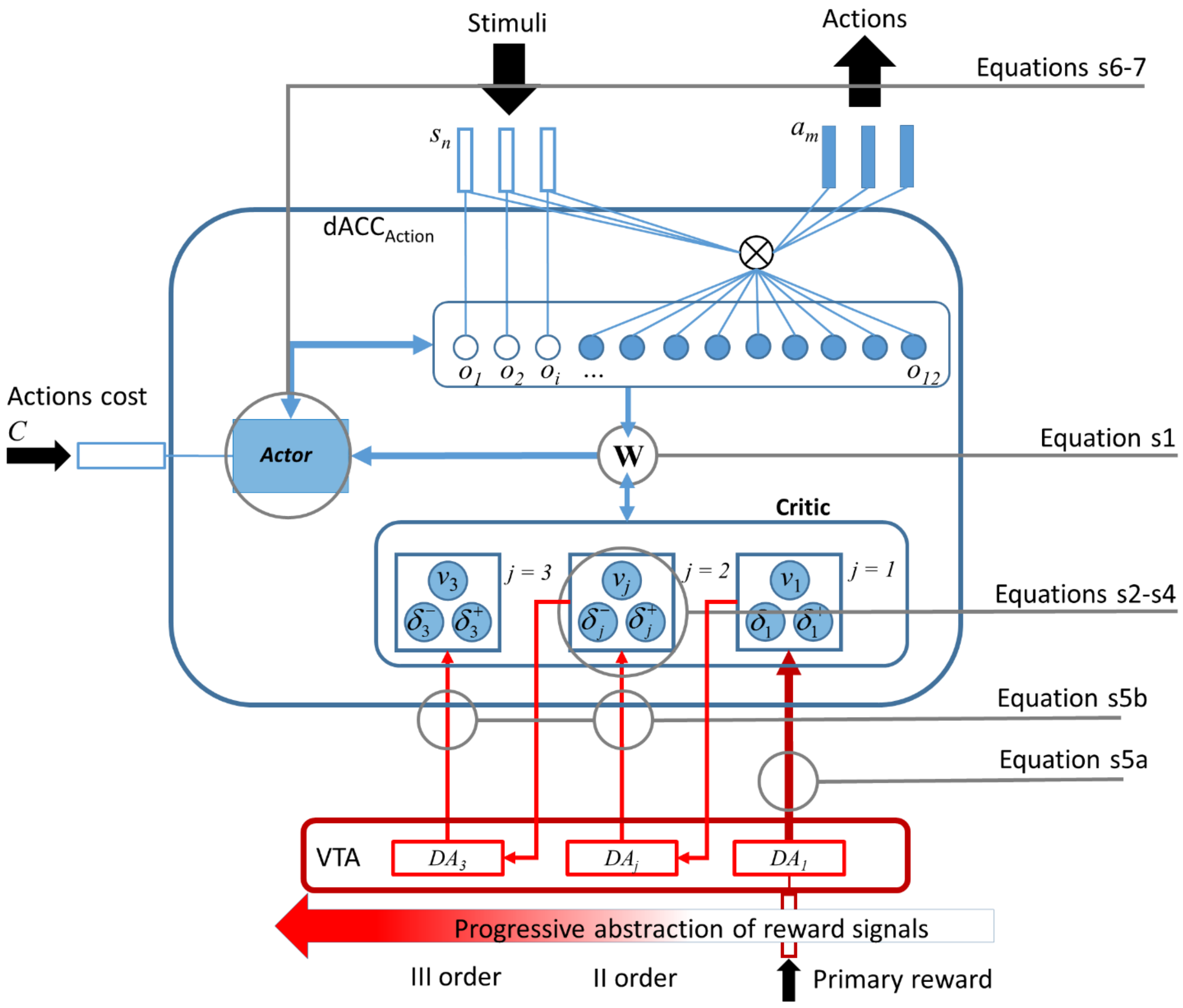

Figure A. Schema displaying in detail the dACC$_{Action}$ – VTA system (see also Figures 1-2 in the main text). Units *o* represent either environmental states (empty disks) or state-action couples (filled disks). The latter are pre-activated by state (**s**) and action (**a**) conjunctions (⊗), then the Actor selects the optimal action based on the Critic evaluations stored in matrix **W**. The reward can be primary (DA_1, dark red arrow), or learned (deriving from higher-order conditioning). In the latter case, after an action selection, the system will perceive a new environmental state (from empty blue bars), which was previously associated to a primary reward. The subsequent activation of the sub-module with j = 1 (which evaluates states and actions from primary rewards) leads to DA_2 activation (arrow from sub-module with j = 1 to DA_2) that rewards the expectations formulated in the sub-module with j = 2, and so on toward a progressive emancipation of learning from primary rewards (bottom gradient arrow).

The dACC-LC system: Learning rate control. As we described also in the main text, we hypothesize that the LC can estimate the Kalman gain by an approximation algorithm based on prediction error and reward prediction signals from the dACC. In Figure B we describe the connection schema between the $dACC_{Act}$ and the LC modules. Each critic sub-module (computing reward prediction and prediction error) is connected to a corresponding LC sub-module, which uses these signals to control the learning rate. The RML approximation of process variance (Var (v)), for the state-action couple o_i, and the j-th critic sub-module, is computed by:

$$\hat{\mathrm{Var}}\left(v(o_i)_{jt}\right) = \left(v(o_i)_{jt} - \hat{v}(o_i)_{jt-1}\right)^2 \quad \text{S8}$$

where $\hat{v}$ is the estimated "true" value of o_i, and it is computed by the following low-pass linear filter:

$$\hat{v}(o_i)_{jt} = \hat{v}(o_i)_{jt-1} + \alpha\left(v(o_i)_{jt} - \hat{v}(o_i)_{jt-1}\right) \quad \text{S9}$$

where α is a meta-parameter ($\alpha = 0.0015$) that defines the frequencies to be filtered out (decreasing α lowers the inferior bound of filtered frequencies). The squared PE in Equation S9 is reported in Equation S8. We now define the estimated global error $\hat{\delta}$ as the estimated unsigned prediction error (that is due to both noise and volatility), and it is computed by:

$$\hat{\delta}(o_i)_{jt} = \hat{\delta}(o_i)_{jt-1} + \alpha\left(\Delta(o_i)_{jt} - \hat{\delta}(o_i)_{jt-1}\right) \qquad \text{S10}$$

where Δ is the instantaneous (at time t) unsigned prediction error:

$$\Delta(o_i)_{jt} = \delta^{+}_{jt} + \delta^{-}_{jt} \qquad \text{S11}$$

Like in Equation S1, if v and δ units are noisy, we need to apply a filter (same threshold θ) to update equations S9-S10 only when v and δ are active above baseline noisy activity. The squared global error ($\hat{\delta}^2$) represents the variance of v due to both process and noise variance. We can now approximate Kalman gain ($\hat{K}$) by:

$$\hat{K}(o_i)_{jt+1} = \frac{\hat{\mathrm{Var}}\left(v(o_i)_{jt}\right)}{\left(\hat{\delta}(o_i)_{jt}\right)^2} \qquad \text{S12}$$

Then, we smooth $\hat{K}$ over time as follows:

$$\hat{K}^s(o_i)_{jt} = \hat{K}^s(o_i)_{jt-1} + \alpha\left(\hat{K}(o_i)_{jt} - \hat{K}^s(o_i)_{jt-1}\right) \qquad \text{S13}$$

And finally we computed the learning rate λ as:

$$\lambda_{jt} = \beta\hat{\overline{K}}^s_{jt} \qquad \text{S14}$$

where $\hat{\bar{K}}^{s}_{jt}$ is the average of $\hat{K}^{s}(o_i)_{jt}$ over vector **o**, and β is a scaling parameter (β = 0.055). We decided to compute one single learning rate for each critic j (averaging over vector **o**), to avoid the strong assumption that the dACC-LC system can provide a specific K estimate for each possible state-action couple. With this algorithm, we are able to approximate Kalman gain with the only assumption that noise power peaks at higher frequency than signal, implemented in the meta-parameter α.

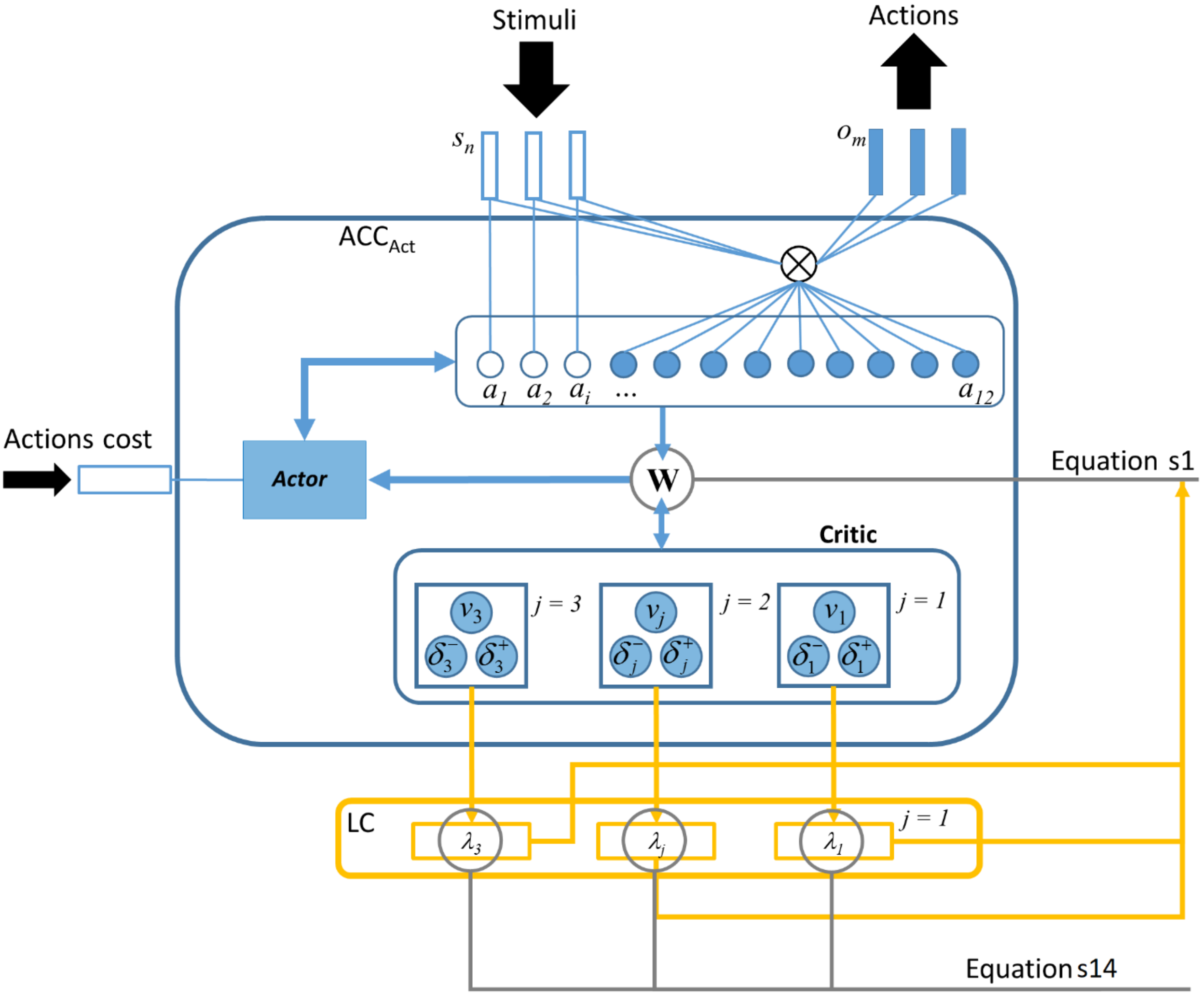

Figure B. Schema of the interactions between the $dACC_{Act}$ module and the LC, for learning rate (λ) control. Each sub-module (indicated with index j) in the dACC is connected with a LC sub-module,

approximating a Kalman filter. This mechanism allows the RML to estimate volatility as a function of reward prediction and prediction error time course (connections from v and δ units), and thence to dynamically change λ, influencing learning dynamics (Equation S1).

The dACC$_{Boost}$-LC-VTA system: cognitive control and catecholamines boosting. In the previous section we described one meta-learning mechanism embedded in the RML, i.e. optimal control of learning rate. RML meta-learning involves also optimal control over primary and nonprimary reward signal (Equation S5a, b) and over cost estimation (Equation S6). We hypothesize that both mechanisms are controlled by an Actor-Critic module (the dACC$_{Boost}$) similar to dACC$_{Act}$, except that the decisions made by the first are directed toward the internal environment (LC and VTA modules). More precisely, the dACC$_{Boost}$ selects the boosting level in order to maximize long-term reward from VTA (Figure C). We assigned to the dACC$_{Boost}$ Critic no index *j* but rather a subscript *B* to distinguish it from the set of critics in the dACC$_{Act}$. The boosting values associated to each specific environmental state are encoded in an n x b matrix (n = 3; b = 10) named **Y**, where rows indicate environmental state (from vector **s**) and columns indicate boosting level (b). Reward signal from VTA is discounted by boosting cost:

$$DA_B = r(R - \omega\, b) \qquad \text{S15}$$

where DA_B is the reward signal from the VTA directed to the dACC$_{Boost}$ module, and $\omega = 0.3$ is a meta-parameter, determining the cost of boosting b. Like in Equation S1, the learning rule is defined by:

$$\dot{q}_{nb} = y_{nb}\lambda_B\left(\delta_B^+ - \delta_B^-\right) \qquad \text{S16}$$

where **Q** is the weights matrix connecting the state-boost units of **Y** to the reward prediction unit v_B in the critic submodule, δ_B are the prediction error units, and λ_B is the corresponding dynamic learning rate. The latter is estimated by means of the approximate Kalman filter algorithm described in Equation S14. All the variables in Equations S8-S14 must be substituted by the variables representing the internal state of the $\text{dACC}_{\text{Boost}}$ module. For example, vector **o** will be substituted by matrix **Y**, and index *j* with subscript *B*. The boosting level *b* is defined by the *b*-th index selected by the Actor according to:

$$p(b \mid \mathbf{q}) = \text{softmax}\left(q_{nb}, \tau\right) \quad \forall n \mid 0 < y_{nb} \qquad \text{S17}$$

Like for the dACC_{Act} module, once the *b*-th boosting unit is selected, all the others are set to zero up to the primary reward onset. Before the selection of *b* by Equation S17, the *y* units that are pre-activated for entering the pool of selectable units (logical condition in Equation S17) are those corresponding at the current environmental state: $y_{nb} = s_n$.

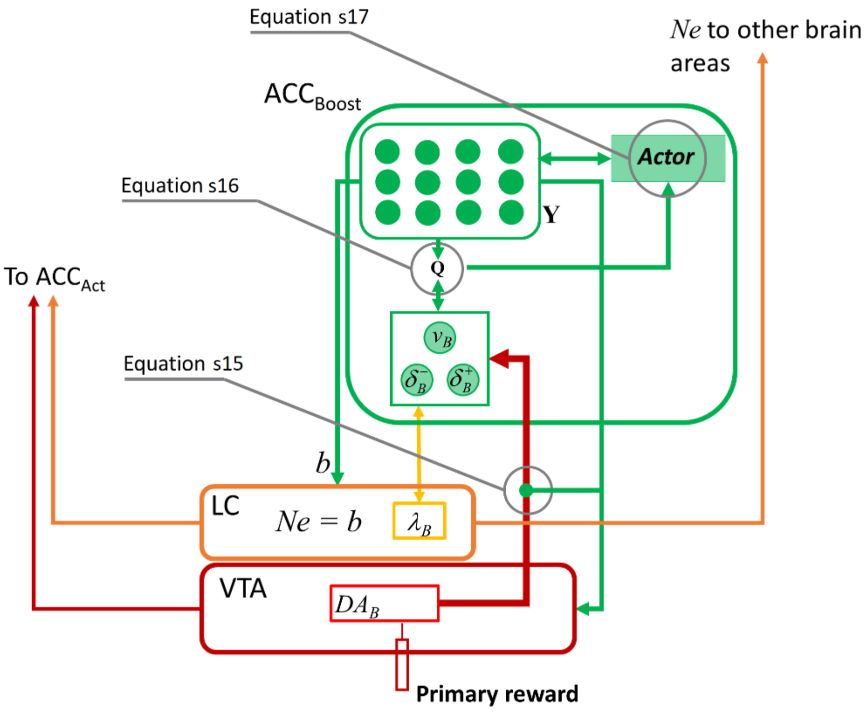

Figure C. dACC$_{Boost}$ module. The machinery of the Actor-Critic system is the same as the one described for the dACC$_{Act}$, except that this system does not select motor actions, but the amount of boosting to increase the level of catecholamines (Equation S17). This system receives only primary rewards discounted by the selected boosting (Equation S15). The aim of this system is to find the optimal boosting (given a specific state *s*) to maximize the net (discounted by boosting cost) long-term primary reward.

Experimental Methods (for Both dynamical and Discrete Model)

General settings. For all the experimental paradigms, environmental state s_3 (arbitrarily chosen) was kept active during each trial, representing a uniform contextual coding of the task. This was not true during higher-order conditioning tasks (simulations 3b-c), where state-to-state transitions were part of the task. For all tasks, every action had a default cost equal to 1 (0.5 for the discrete model; this is a general cost of emitting a response), except for the "stay" option which had a cost equal to zero (as in this case the RML decides to emit no response). "Stay" option was never rewarded, when the RML decided to "Stay", the stimulus delivering program simply moved to the next trial.

Task settings were the same for both dynamical and discrete model, with exception of simulations 2a-c (see below). All the initial state-action values for the dACC modules were set to zero, while initial value for the learning rate (λ) was 0.003 for the dynamical model and .3 for the discrete model.

Simulations 1 and S1 (learning rate control). The task, described in Figure 2a-b (main text), was administered in a version where the outcome was binary (Simulation 1, main text) and in a version where the outcome was continuous (Simulation S1, below).

Simulation 1. For the binary task, the reward rates and magnitudes for respectively optimal and non-optimal choices are reported in Table B. We assigned higher reward magnitudes to choices with lower reward rates [4], in order to promote switching between choices and to make the task more challenging. The Pascalian value of each choice (probability × magnitude) remained higher for higher reward rates.

Table B. Mean reward rates assigned to the 2 possible choices in the task of Simulation 1, and their respective magnitudes (in arbitrary values).

Statistical condition	Reward rate	Reward magnitude
Stat (stationary)	70%-30%	1.5-2.5
Stat2 (stationary uncertain)	60%-60%	2-2
Vol (volatile)	90%-10%	1.5-2.5

We ran 12 simulations, reproducing the performance of 12 experimental subjects. The order of presentation of different statistical environment was randomized between subjects. Yet like in

Behrens et al. (2007), each simulated subject executed one Stat condition (practice) before the task. Each statistical condition consisted of 144 trials, for a total of 432 trials. All the statistical analyses were conducted after excluding the first 20 trials from each statistical condition, in order to exclude the influence of volatility effects at the transitions between stationary environments (like in Behrens et al., 2007; Silvetti et al., 2013). In the volatile (Vol) condition, the reward rate (and associated reward magnitude) switched between options every 18 trials on average (uniform distribution between 14 and 22 trials).

Simulation S1. For the continuous task version, the reward rate was the same for every option (80%) but reward magnitude varied trial-by-trial with a random Gaussian distribution (Table C). All the other features were in common with the binary task, except for the fact that volatility was introduced by switching exclusively mean reward magnitudes between choices.

Table C. Simulation S1, task specifics

Statistical condition	Mean reward magnitude	Reward magnitude variance
Stat (stationary)	2-1	0.04-0.04
Stat2 (stationary uncertain)	2-2	2.25-2.25
Vol (volatile)	3-1	0.04-0.04

Mean reward magnitudes assigned to the 2 possible choices in the task of Simulation 1b (arbitrary values), and their respective variances (e.g., to choice 1 in Stat condition was assigned a mean reward magnitude of 2, with variance 0.04). In Vol condition, the two mean reward magnitudes switched between choices, on average every 18 trials.

Simulation 2a: Physical effort control and decision-making in challenging cost/benefit trade off conditions. The task was executed both in normal conditions and after DA lesion. Before task execution, we administered to the RML a version of the Effort task where both the possible options

required low effort, to expose the model to different reward magnitudes [6]. For the DA lesion condition, we first administered the No Effort task to the normal RML, then we implemented the DA lesion and afterwards we administered the Effort task. The lesion to the dopaminergic system was simulated multiplying all the VTA outputs by 0.6 (40% lesion; 70% lesion for the discrete version of the model), while the lesion to both the dACC modules was simulated by multiplying the activity of all the neural units (including the boosting signal efferent from the $dACC_{Boost}$ module) by 0.6 (0.7 for the discrete model). Each experiment consisted of 70 trials. We summarize costs and rewards magnitudes in Table D. All the statistical analyses were run on the last 40 trials, in order to rule out learning effects. Animal data are from reference [6], for DA lesion we averaged the behavioural results from both haloperidol administration and nucleus accumbens lesion from Figures 3-5 in [6]. Data extraction from figures was performed by WebPlotDigitizer.

Table D. Summary of effort-to-reward ratios for all the option type in tasks administered in Simulations 2a and 2b.

Effort/reward	High reward	Low reward
**High effort**	7/3 (6/5)	7/1 (6/1)
**Low effort**	1/3 (0.5/5)	1/1 (0.5/1)

Values used for the discrete version of the model are between brackets.

The net subjective value (*nsv*) represented in Figure 4g was computed as the sum of the net values from both the dACC modules:

$$nsv = h_i + q_{nb} \tag{S18}$$

where h and q are the net estimated values from respectively $dACC_{Act}$ (Equation S6) and $dACC_{Boost}$ (Equation S16).

Simulation 2b: performance recovery after DA lesion, in cost/benefit trade off conditions. We tested the potential recovery of the preference for high reward option, by administering to the DA lesion group two other tasks where effort differences were removed. To the DA lesion group, after the Effort task execution, we administered either a No Effort task (Figure 4d, two low effort options) or a Double Effort task (Figure 6a, two high effort options). Table D summarizes the effort-to-reward ratios also for these tasks. Animal data for comparison are from [6], we averaged the behavioural results across the experimental blocks in Figure 5 (for Double Effort and No Effort respectively) from [6]. Data extraction from original figures was performed by WebPlotDigitizer.

Simulation 2c: Adapting cognitive effort in a WM task (only dynamical model). *FROST model description*. We implemented a simplified version of FROST (FROntal-Striatal-Thalamic) model [7] to model the WM module. We did not implement the subcortical modules of this model, as we were not interested in simulating in detail the whole neural dynamics in WM-related circuits. Our implementation of the FROST model consisted of a dynamical system of two differential equations (fronto-parietal recurrent network), which updates the activation of each single neuron within a neural matrix of size 3 × 4 representing a 2D space. The first equation models the posterior parietal cortex (P) dynamics:

$$\dot{P}_{ij} = \varphi I_{ij}\left(\varphi - P_{ij}\right) + \psi F_{ij}\left(1 - P_{ij}\right) - \xi P_{ij} \qquad \text{S19}$$

where I_{ij} is the visual input from the ij-th visual unit. The activation of I is transient and encodes the presence of a visual stimulus. F_{ij} is the activation of the ij-th unit in the frontal cortex. Parameters take on the following values: $\varphi = 0.4$, $\psi = 0.01$, $\xi = 0.01$. The dynamics of prefrontal neurons (F) is defined by:

$$\dot{F}_{ij} = \left(\varpi A_{ij} + \psi P_{ij}\right)\left(1 - F_{ij}\right) - \zeta NE^{-1}\left(\sum_{k\neq i,l\neq j} F_{kl}\right)F_{ij} - \upsilon F_{ij} \qquad \text{S20}$$

where *A* is a signal gating WM retention. It assumed value equal to 1 for the time window when the WM has to retain information, otherwise it is zero. In Ashby et al. (2005), *A* is defined by cortical-subcortical interactions, in our simulation it was turned to 1 at the beginning of each trial and back to zero after the match/mismatch response by the RML. There are three parameters with the following values: ϖ = 0.005, ζ = 0.1, υ = 0.02. The term $NE^{-1}\left(\sum_{k\neq i,l\neq j} F_{kl}\right)F_{ij}$ models lateral inhibition between prefrontal neurons. This means that lateral inhibition increases when memory load increases. This term is modulated by LC input to the prefrontal cortex (*NE*), such as the higher the LC input the lower is lateral inhibition effect. Therefore, LC input causes a better representation of items in WM (gain modulation), increasing the activation especially in those neurons that are encoding item positions (Figure D).

RML-FROST interface. The RML selects the match-mismatch option by Equation S7 biased by FROST frontal neural representations. More specifically, FROST frontal neurons modulate vector **h** in Equation S7 as follows:

$$h^{*}_{match} = h_{match} + \alpha_F \max\left(\left[F_{ij} - \theta_{F1}\right]^{+}\right) \qquad \text{S21}$$

$$h^{*}_{mismatch} = h_{mismatch} + \alpha_F \left[\left\langle F_{ij}\right\rangle - \theta_{F2}\right]^{+} \qquad \text{S22}$$

where $\mathbf{h}^*$ is the modulated vector for matching and mismatching options while $\alpha_F = 17$, $\theta_{F1} = 0.22$ and $\theta_{F2} = 0.12$ are parameters. Such a modulation contrasts the average frontal network activation ($\langle F_{ij} \rangle$ in Equation S22, mismatch) against the activation of the neural unit with the maximal activation (Equation S21, match). Afterwards, vector $\mathbf{h}^*$ is passed as an argument (replacing $\mathbf{h}$) to Equation S7 for action selection. It is relevant to note that in this task, action selection would depend on both activity of frontal neurons and on action values (stored in $\mathbf{h}$). Because both match and mismatch responses were rewarded with the same reward magnitude and they had the same action cost C, frontal activity was the only factor biasing the response.

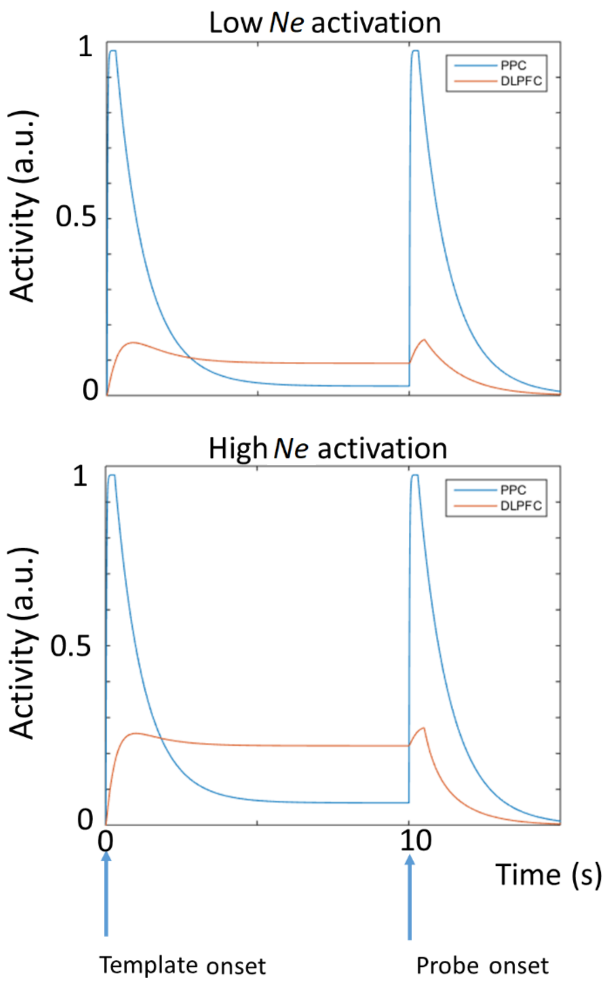

Figure D. Activity of two neural units that are encoding one item during a trial. Red plot: prefrontal cortex (DLPFC) unit activation; blue plot: posterior parietal cortex (PPC) unit activation. After template

presentation (delay between template onset and probe onset, cfr Figure 7a, main text), information about items position is retained without perceptual input (recurrent PPC-DLPFC dynamics). LC input (*NE*) to prefrontal neurons increases the activation only for those neurons that were already active to code for template items.

Simulations 3a-b. VTA activity plotted in Figure 8 was represented by the time course of DA_j with j >1 (Equation S5b).

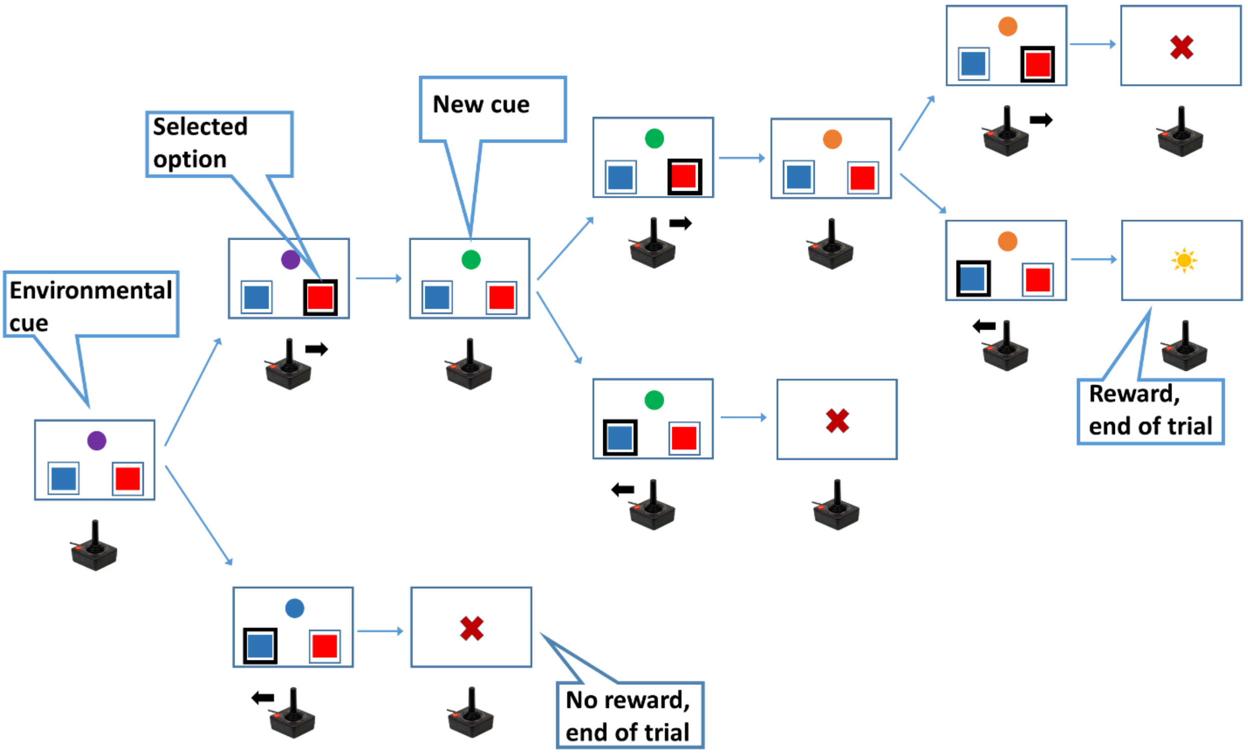

Figure E. Possible bifurcations during a trial of higher-order instrumental conditioning task.

Simulation S2 (DA shifting during classical conditioning; only dynamical model). We administered a classical conditioning task, where an environmental cue lasted for 2s, followed by a primary reward on 80% of all trials. Inter trial interval was 4s. The model was trained with 40 trials for each simulation, for each of 12 simulations (subjects).

References

1. Silvetti M, Seurinck R, Verguts T. Value and prediction error estimation account for volatility effects in ACC: A model-based fMRI study. Cortex. 2013; doi:10.1016/j.cortex.2012.05.008

2. Cohen JY, Haesler S, Vong L, Lowell BB, Uchida N. Neuron-type-specific signals for reward and punishment in the ventral tegmental area. Nature. 2012;482: 85–88. doi:10.1038/nature10754

3. Takikawa Y, Kawagoe R, Hikosaka O. A possible role of midbrain dopamine neurons in short- and long-term adaptation of saccades to position-reward mapping. J Neurophysiol. 2004;92: 2520–9. doi:10.1152/jn.00238.2004

4. Behrens TE, Woolrich MW, Walton ME, Rushworth MF. Learning the value of information in an uncertain world. Nat Neurosci. 2007;10: 1214–1221.

5. Silvetti M, Seurinck R, van Bochove ME, Verguts T. The influence of the noradrenergic system on optimal control of neural plasticity. Front Behav Neurosci. 2013;in press: 160. doi:10.3389/fnbeh.2013.00160

6. Walton ME, Groves J, Jennings KA, Croxson PL, Sharp T, Rushworth MFS, et al. Comparing the role of the anterior cingulate cortex and 6-hydroxydopamine nucleus accumbens lesions on operant effort-based decision making. Eur J Neurosci. 2009;29: 1678–1691. doi:10.1111/j.1460-9568.2009.06726.x

7. Ashby FG, Ell SW, Valentin V V., Casale MB. FROST: A Distributed Neurocomputational Model of Working Memory Maintenance. J Cogn Neurosci. 2005;17: 1728–1743. doi:10.1162/089892905774589271
