# Supplementary material for: Dorsal anterior cingulate-brainstem ensemble as a reinforcement meta-learner
Source: PLoS Comput Biol. 2018 Aug 24;14(8):e1006370. doi: 10.1371/journal.pcbi.1006370 (PMC6126878; doi:10.1371/journal.pcbi.1006370)
Supplement: S2 File — (PDF) [file pcbi.1006370.s002.pdf]

Supplementary Results: Dynamical Model

Simulation S1 (learning rate control).

In the continuous version of the task administered in Simulation 1, the RML performance in terms of optimal choices percentages was: Stat = 80% (± 1.5% s.e.m.), Vol = 61% (± 2.6% s.e.m.). The percentage of optimal choices in the Stat condition is higher for the continuous task than for the binary one. This is because in Simulation 1, we made decisions more challenging by assigning a slightly higher reward magnitude to options with a lower reward probability (see Table B in S1 File). Learning rate control as a function of volatility did not change with respect to the binary version of the task (Figure F). Also for the continuous task version, there was a main effect of volatility on learning rate ($F(2,11) = 15.3$, $p = 0.0001$). Post-hoc analysis shows that stationary conditions did not differ (Stat2 > Stat, $t(11) = 2$, $p = 0.07$), while in volatile condition learning rate was higher than in stationary conditions (Vol > Stat2, $t(11) = 3.47$, $p < 0.005$; Vol > Stat, $t(11) = 5.2$, $p < 0.0001$).

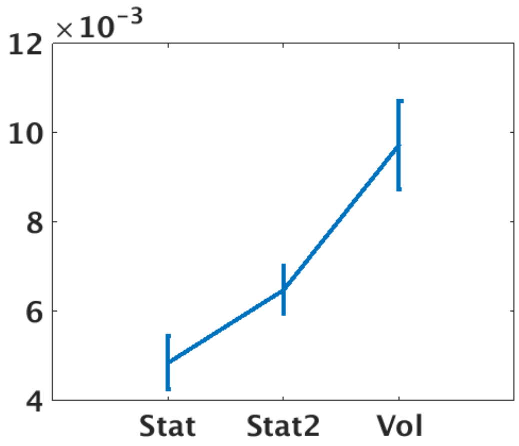

Figure F Learning rate (± s.e.m.) as a function of environmental volatility in the continuous version of the task in Simulation S1.

Simulation S2: DA Shifting in Classical Conditioning.

A typical experimental finding on DA dynamics is the progressive shifting of DA release onset from primary reward to CS [1]. At the same time, omission of expected primary reward typically leads to dips in neural activity in dopaminergic neurons, dropping their discharge rate to zero. DA shifting develops exclusively in the CS-locked and US-locked time windows, without the signal progressively propagating backward from US to CS [1]. We now investigate these properties in the RML.

Simulation results and discussion. Figure G shows the VTA response (both from RML and animal data) during a classical conditioning paradigm. These results replicate our previous model RVPM simulations (Silvetti et al. 2011). We hypothesized (cf Equation S5b) that DA dynamics during conditional learning is determined by dACC-VTA interaction, by combining the information from reward expectation and reward PE. More precisely, cue-locked VTA activity shown in Figure Gc-d is due to the reward prediction temporal difference ($[\dot{v}]^{+}$), while reward-locked activity is due to PE temporal difference ($\left[\dot{\delta}^{+}\right]^{+}-\left[\dot{\delta}^{-}\right]^{+}$). This mechanism can closely simulate the progressive shifting of DA activity from reward period to cue period (Figure Gc), and the DA dip when expected rewards are omitted (Figure Gd). In conclusion, we propose that higher-order conditioning (in instrumental paradigms: Simulation 3b) is based on the DA response due to CS-locked reward prediction signal from the dACC.

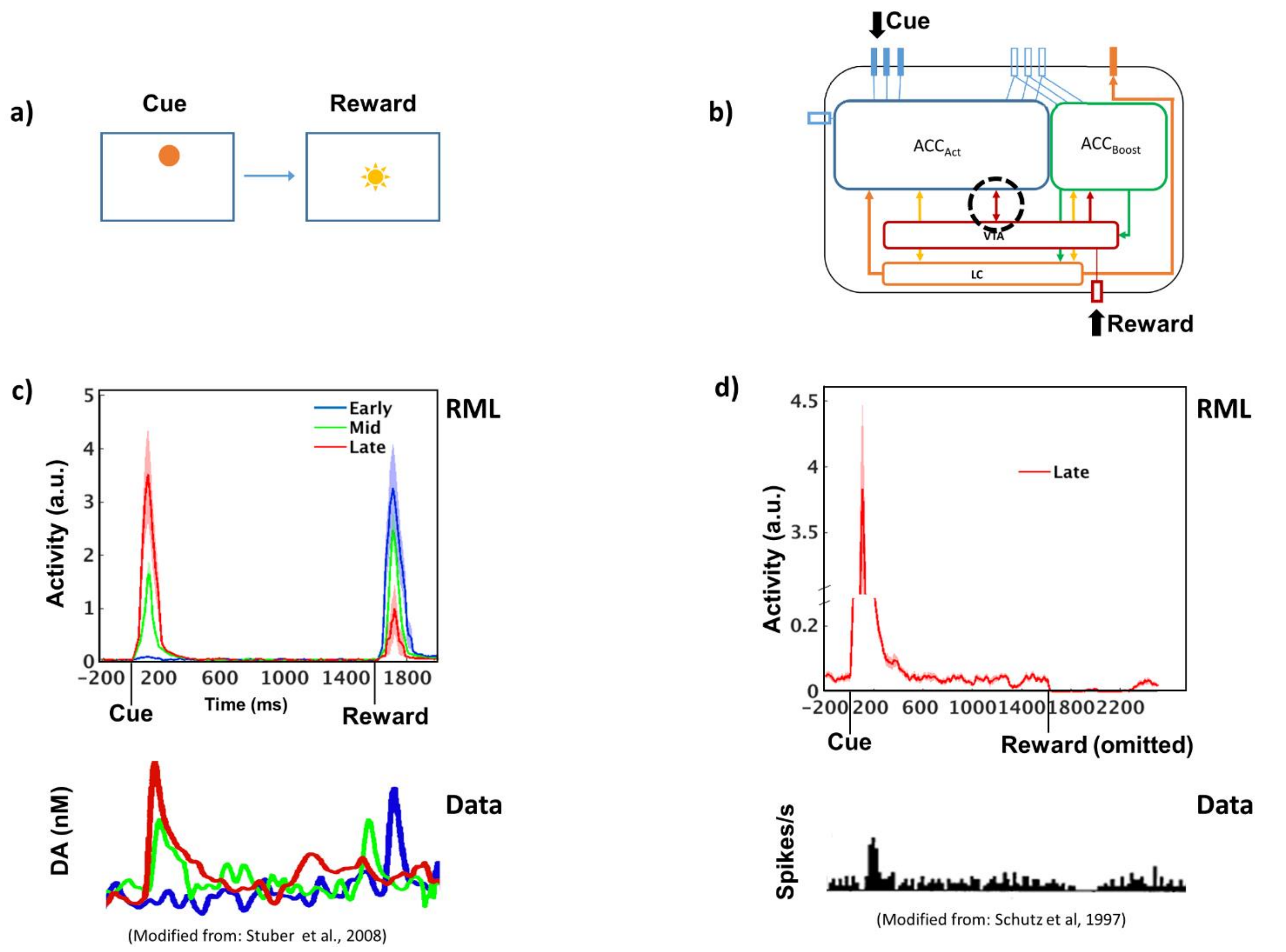

Figure G. Dynamical version of RML. **a)** Classical conditioning task administered to the model. A cue was presented, then a primary reward was delivered. **b)** Recording site (dashed circle) of VTA activity (Equation S5b) plotted in c and d. Black arrows indicate cue and reward input to the RML. **c)** Simulated VTA activity shifting from reward to cue period in three different training phases (early, mid and late). The bottom plot shows empirical data from Stuber et al. (2008). **d)** Simulated VTA baseline activity suppression when an expected reward is omitted after extensive conditional training (late training phase). The bottom plot shows empirical data from Schultz et al. (1997).

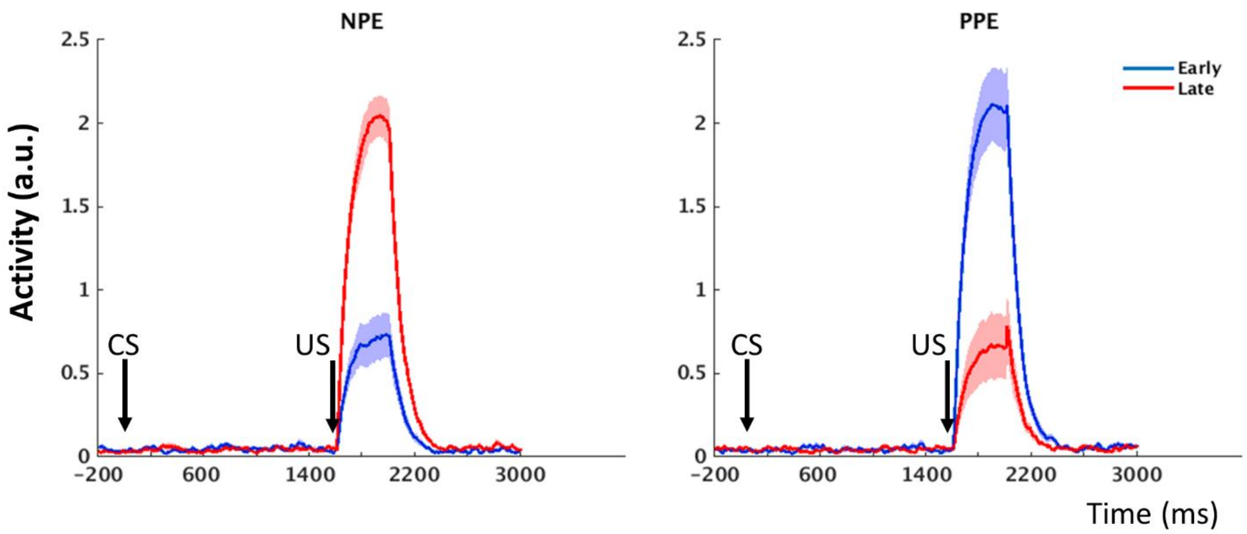

Figure H. Dynamical version of RML $dACC_{Act}$ module prediction error activity during classical conditioning, in early and late stages of training. NPE: reward-locked (US) negative prediction error time course grand average (in missed reward trials). PPE: reward-locked positive prediction error time course grand average (in rewarded trials). Plot shadows indicate s.e.m., plots are recorded from δ^+ (PPE) and δ^- (NPE) units (Equations S3-4) in the Critic's sub-module with j = 1 (computing first-order conditioning).

Supplementary Results: Main results replication with the discrete model

Simulation S3: Learning rate control (replication of Simulations 1 and S1 with the discrete model)

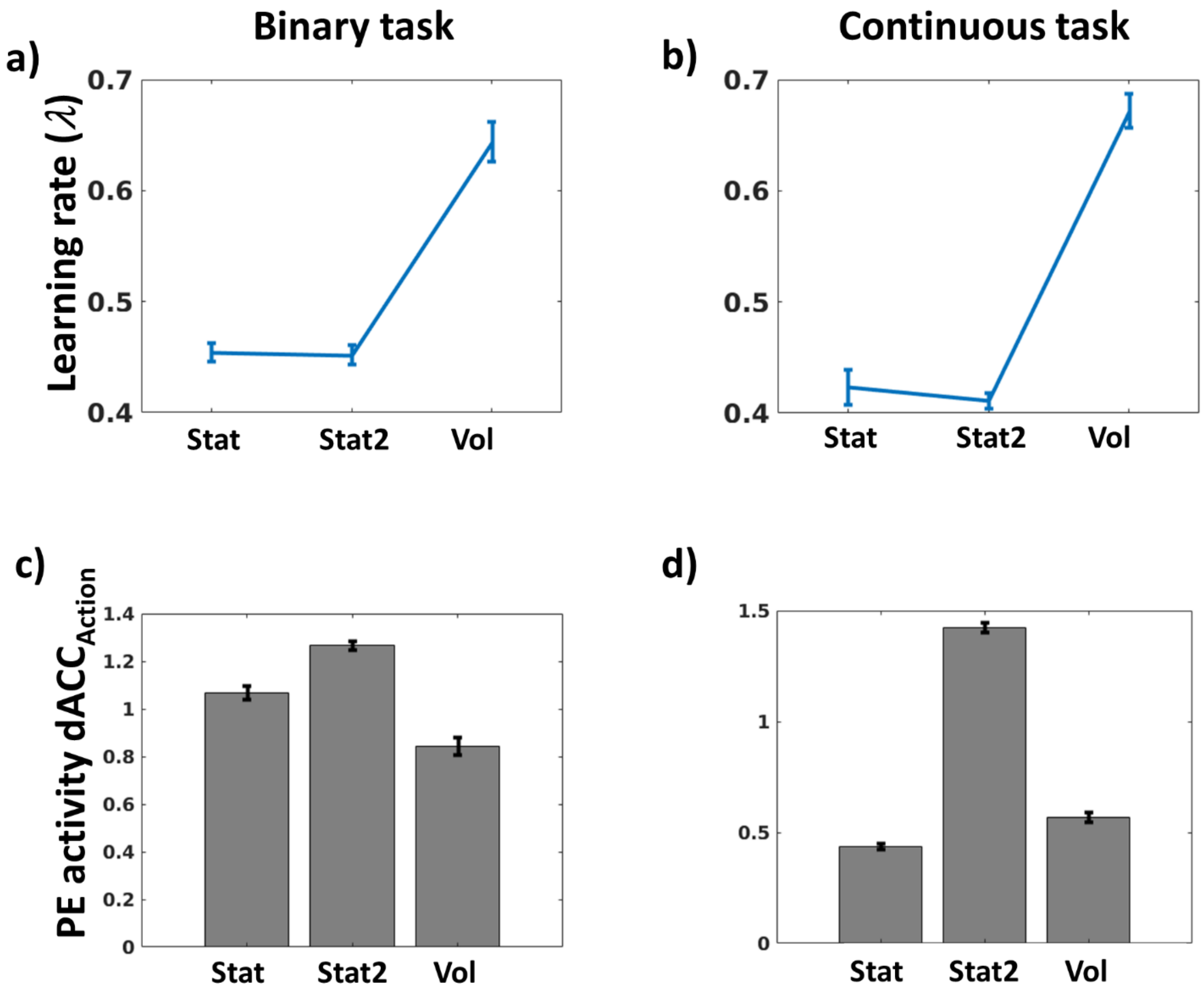

Figure I. Discrete version of RML. **a)** Learning rate λ (± s.e.m.) as a function of environmental volatility in the binary version of the task, to be compared with Figure 2 (main text) from the dynamical RML. **b)** Learning rate λ (± s.e.m.) as a function of environmental volatility in the continuous version of the task, to be compared with Figure F from the dynamical RML. **c-d)** Unsigned PE activity recorded from $dACC_{Act}$ module as a function of environmental volatility (± s.e.m.), during respectively binary and the continuous version of the task. In both cases, dACC responded more strongly to environmental entropy (Stat2 condition) rather than to volatility. Plots

can be compared with the one in Figure 3b (main text), which is from the dynamical version of the model.

Simulation S4a: physical effort optimization (replication of Simulation 2a with the discrete model)

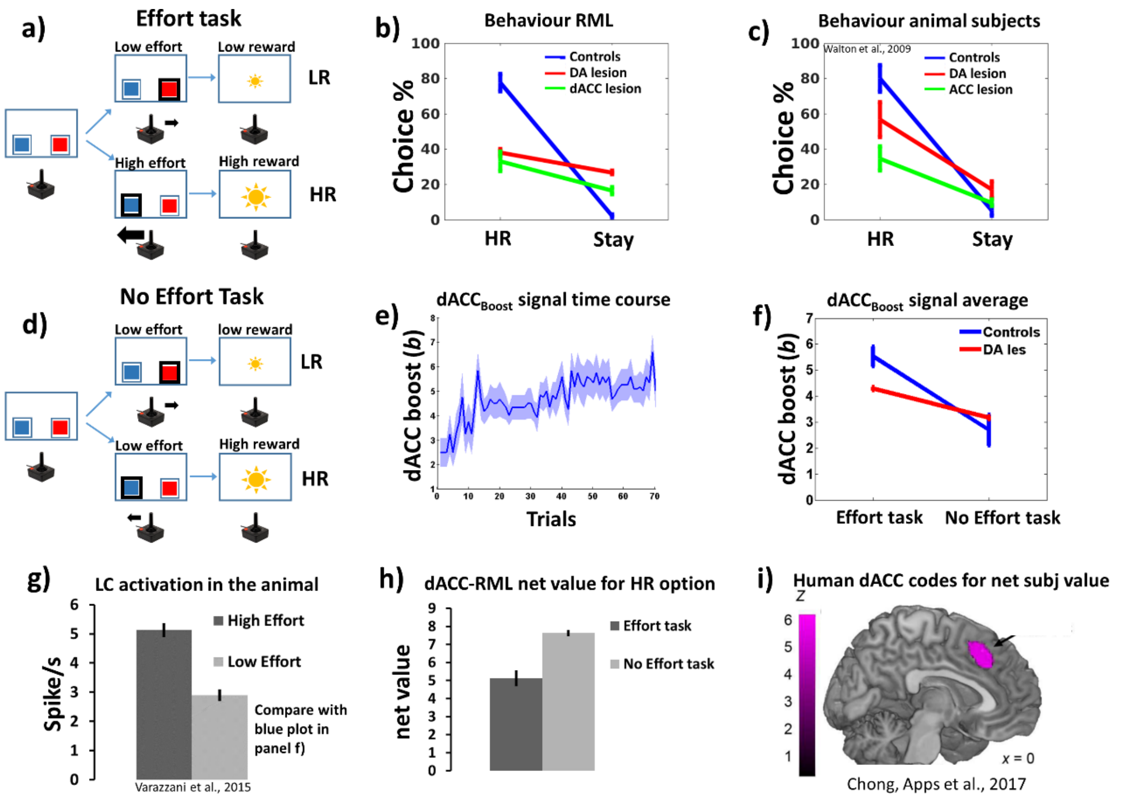

Figure J. Discrete version of RML. Effort task results obtained with the discrete version of the model, to be compared with Figure 4 (main text; same results obtained with the dynamical model), with which it shares the same caption. Some s.e.m. intervals are not visible due to small variance.

Simulation S4b: physical effort optimization (replication of Simulation 2b with the discrete model)

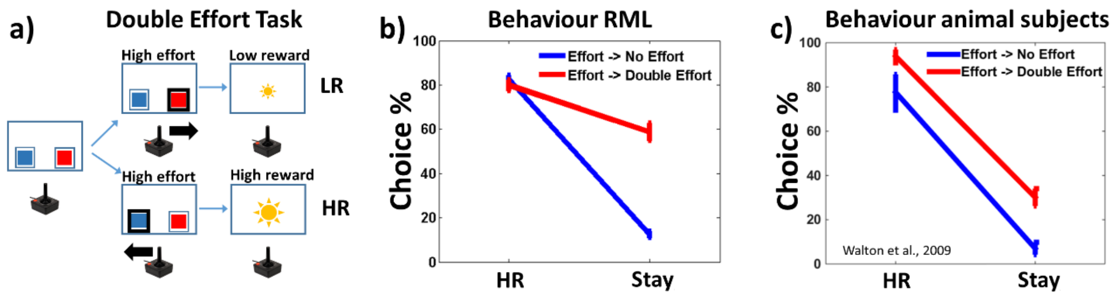

Figure K. Discrete version of RML. Recovery of HR option preference in DA lesioned subjects, results obtained with the discrete form of the model. This figure is to compare with Figure 6 (main text; same experiment with dynamical model), with which it shares the caption. Some error intervals are not visible due to small variance.

Simulation S5a and S5b: classical and instrumental higher-order conditioning (replication of simulations 3a-b with the discrete model)

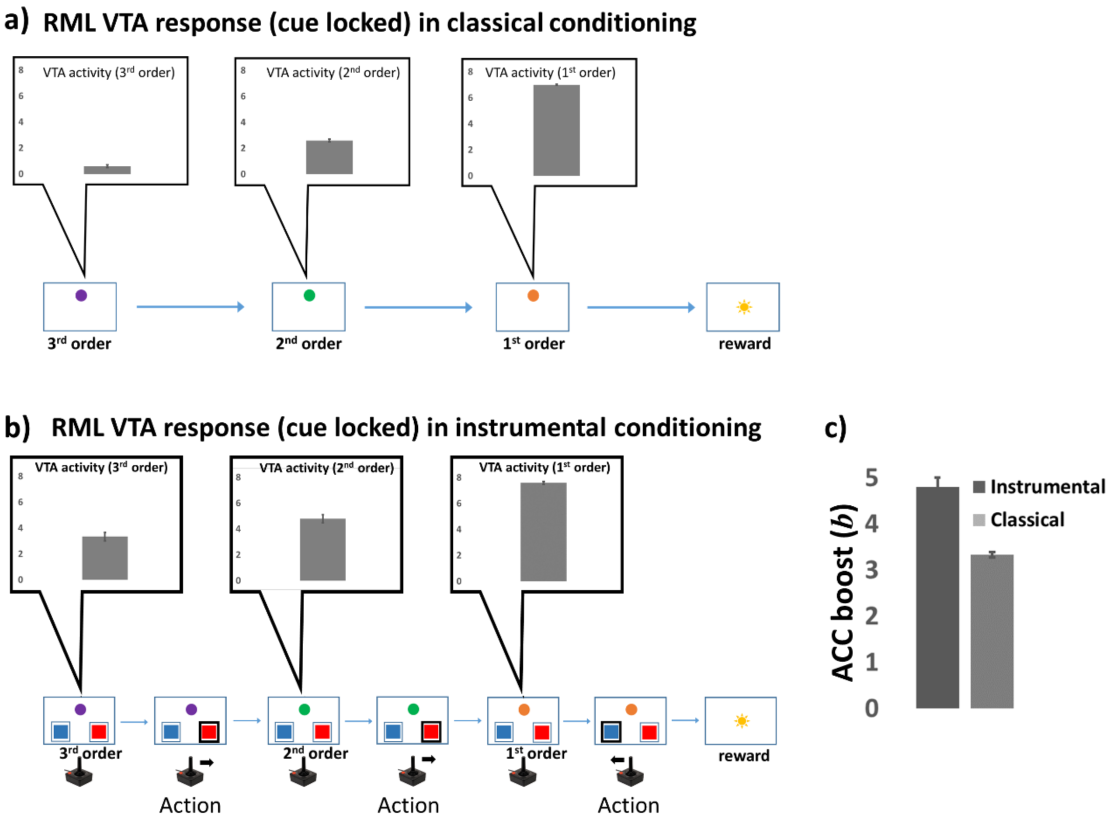

Figure L. Discrete version of RML. **a)** Experimental results for higher-order classical conditioning. **b)** Experimental results for higher-order instrumental conditioning. **c)** $dACC_{Boost}$ efferent signal as a function of conditioning paradigm. This figure is to be compared with Figure 8 (main text; same experiment with dynamical model), with which it shares the caption. VTA activity is from Equation 6a (main text).

References

1. Schultz W, Apicella P, Ljungberg T. Responses of monkey dopamine neurons to reward and conditioned stimuli during successive steps of learning a delayed response task. J Neurosci. 1993;13: 900–913.

2. Silvetti M, Seurinck R, Verguts T. Value and prediction error in medial frontal cortex: integrating the single-unit and systems levels of analysis. Front Hum Neurosci. 2011;5: 75. doi:10.3389/fnhum.2011.00075

3. Stuber GD, Klanker M, de Ridder B, Bowers MS, Joosten RN, Feenstra MG, et al. Reward-predictive cues enhance excitatory synaptic strength onto midbrain dopamine neurons. Science (80-). 2008;321: 1690–1692.

4. Schultz W, Dayan P, Montague PR. A neural substrate of prediction and reward. Science (80-). 1997;275: 1593–1599.
